# Unlocking the power of *Pseudomonas*: network-guided isolation enhances plant growth on *Achnatherum inebrians*

**DOI:** 10.1128/msystems.00563-26

**Published:** 2026-09-02

**Authors:** Jinjin Liang, Chunjie Li, Xingxu Zhang, Zhibiao Nan

**Affiliations:** 1 State Key Laboratory of Herbage Improvement and Grassland Agro-ecosystems, College of Pastoral Agriculture Science and Technology, Lanzhou University 655661, Lanzhou, China; University of Wisconsin-Madison, Madison, Wisconsin, USA

**Keywords:** network analysis, culture-based isolation, seed-associated, phyllosphere, rhizosphere soil, *Epichloë *endophyte, *Pseudomonas*, keystone taxa

## Abstract

The tripartite interplay among plants, bacteria, and fungal endophytes is crucial for maintaining host plant fitness. However, how *Epichloë* endophytes influence plant-associated bacterial communities in cool-season grasses, particularly in ecologically important yet understudied species, such as *Achnatherum inebrians*, remains unclear. Using phylogenetic molecular ecological network analysis, we determined that seed-borne (seed epiphytic) and phyllosphere bacterial communities of *Epichloë*-infected (EI) *A. inebrians* exhibited reduced network complexity compared to *Epichloë*-free (EF) plants. Across all samples, Proteobacteria and Firmicutes dominated the keystone taxa, with *Pseudomonas* (OTU744 and OTU8264) consistently identified as a hub genus in both seed-borne and phyllosphere networks. Culture-based analysis revealed that endophyte-infected plants had a significantly (*P* < 0.05) higher relative abundance of *Pseudomonas* and *Bacillus* than EF *A. inebrians*, especially *Pseudomonas* comprised 13, 35, and 33% of isolates from the seed, leaf, and rhizosphere of *A. inebrians*, respectively. To capture potential functional diversity, we selected two phylogenetically distant *Pseudomonas* strains from each of the three ecological niches for further analysis. Inoculation of *A. inebrians* seedlings with these strains consistently promoted plant growth, enhanced forage quality (total nitrogen content), and improved nutritional value (ether extract). Whole-genome sequencing combined core-genome phylogenetic tree of the six *Pseudomonas* strains and confirmed that five strains belong to *P. atacamensis*, whereas Pse19 was *P. cucumis*. Our findings reveal that *Epichloë* endophytes modulate bacterial network stability and enrich plant-associated *Pseudomonas*, which synergistically enhance host performance. Collectively, this study provides a mechanistic framework for manipulating keystone taxa and beneficial isolates to improve grass productivity in grassland agricultural ecosystems.

**IMPORTANCE:**

Although the tripartite interplay between plants, bacteria, and fungal endophytes, such as *Epichloë*, is recognized as vital for host fitness, the specific mechanisms through which these endophytes shape associated bacterial communities, particularly in ecologically significant grasses, such as *Achnatherum inebrians*, remain poorly understood. This study provides crucial mechanistic insights by revealing that *Epichloë* endophytes reconfigure the structure and stability of both seed-borne and phyllosphere bacterial networks in *A. inebrians*, leading to reduced complexity but enrichment of specific keystone taxa. We identify *Pseudomonas* as a consistently dominant hub genus across these niches. Notably, functional validation shows that diverse *Pseudomonas* isolates, representative of those enriched by the endophyte, significantly enhance host plant growth, forage quality, and nutritional value. Our findings reveal a synergistic mechanism where *Epichloë* endophytes modulate bacterial network stability to favor beneficial *Pseudomonas* populations, collectively boosting host performance.

## INTRODUCTION

*Achnatherum inebrians* (also known as drunken horse grass) is a perennial species that is becoming increasingly widespread in the highly modified arid and semi-arid grasslands of northwest China (1, 2). Nearly all wild *A. inebrians* host a seed-borne, mutualistic fungal endophyte, either *Epichloë gansuensis* (2, 3) or *E. inebrians* (4). The presence of *Epichloë* endophytes enhances tolerance to various biotic stresses, such as insect pests (5) and pathogenic fungi (6), as well as abiotic stresses, including heavy metals (7), low temperatures (8), salinity (9), drought (10), low nitrogen (N) (11), and low phosphorus (*P*) (12). *A. inebrians* has the potential for ecological remediation owing to its remarkable ability to survive in harsh conditions. In addition, it is an important forage grass, as *Epichloë*-free grass can be safely consumed by livestock and herbivores (13).

Plants in natural ecosystems are ‌closely associated with a diverse range of microorganisms, including bacteria, fungi, archaea, protists, and viruses, collectively known as the plant-associated microbiome (14, 15). These microorganisms can be beneficial, harmful, or neutral, and they play crucial roles in plant growth and development (16). In particular, beneficial interactions between host plants and microorganisms are bidirectional. Plants release up to 100 molecules derived from their photosynthetic carbon sources through root systems to communicate with bacteria and fungi (17). Conversely, these microorganisms interact directly with plants, particularly in the rhizosphere, by colonizing roots to form symbiotic relationships with the plant, such as mycorrhizal associations, or by promoting plant growth through phytohormone production (18). Studies have shown that Gramineae plants interact with their associated microbiome, including seed-associated, phyllosphere, and rhizosphere microbes (19). These microorganisms enhance abiotic stress tolerance (18, 20), improve nutrient acquisition (21), and provide protection against plant pathogens (22, 23). Notably, the rhizosphere microbiome is widely recognized as the second genome of the plant and plays a critical role in host growth and health (24).

Research methods for studying microorganisms primarily include culture isolation, high-throughput amplicon sequencing, and metagenomic sequencing. Cultural isolation is essential for understanding the functional roles of microorganisms in plant growth and health. By combining isolated and cultured microorganisms with sterile plant culture systems, researchers can elucidate causal relationships and mechanisms of interactions between plant-associated microbiomes and plant growth phenotypes. This approach represents a key technological advancement, shifting plant-associated microbiome research from descriptive to functional studies (25, 26). Amplicon sequencing and shotgun metagenomic approaches have also been used to characterize the microbial diversity and functional potential of plant-associated microbial communities across diverse plant species (27). Microbial network analysis based on these sequencing methods has become a crucial tool for visualizing co-occurrence patterns within microbial communities using high-throughput data (28, 29). Keystone species within these networks can be identified by their capacity to significantly alter microbiome structure and function (30). Previous studies have integrated network analysis with culturing techniques to identify and inoculate microorganisms, thereby validating keystone and core microbes of host plants. These studies further reveal that such microorganisms exert critical effects on host plants (31, 32).

*Pseudomonas*, a genus within the phylum Pseudomonadota, order Pseudomonadales, and family Pseudomonadaceae, is a key group of plant growth-promoting rhizobacteria (PGPR). These bacteria extensively colonize plant-associated niches and interact with hosts to support their growth and health (33, 34). To date, more than 300 *Pseudomonas* species have been identified (35). *Pseudomonas* contributes to plant health and growth by participating in nutrient cycling through phosphate solubilization (36), producing phytohormones that stimulate root growth (37), synthesizing antibacterial compounds, such as lipopeptides, to outcompete pathogens for resources (38), and forming biofilms or recruiting beneficial microbes in the rhizosphere to improve nutrient absorption and enhance resistance to root diseases (39).

Previous studies on the interactions between *A. inebrians* and its microbiome have primarily focused on the composition and diversity of microbial communities in the rhizosphere (40–42), phyllosphere (43), and seed-borne niches (44), as well as the effects of rhizosphere growth-promoting bacteria on host growth and development (41, 45). However, systematic analyses of *A. inebrians*-associated microorganisms integrating network analysis with culturing methods are lacking. In addition, the relationship between keystone species identified through network analysis and isolated microbial strains, as well as the functional validation of these relationships in *A. inebrians*, remains largely unexplored. To date, *Pseudomonas* has been detected only in the roots and leaves of *A. inebrians*, with nearly 30 isolated *Pseudomonas* strains shown to have positive effects on the host (41, 45).

Although our previous studies characterized the microbial community composition across different niches of *A. inebrians* and identified several beneficial *Pseudomonas* strains, it remains unclear whether culturable isolates correspond to ecologically important keystone taxa associated with microbial community structure and stability. This study integrated phylogenetic molecular ecological network analysis, culture-dependent isolation, and plant inoculation assays to investigate the relationships among *Epichloë* endophytes, plant-associated bacterial communities, and host performance. We hypothesized that *Epichloë* endophyte infection alters the assembly of the bacterial microbiome and promotes the enrichment of beneficial keystone taxa, particularly *Pseudomonas*, which may contribute to host plant growth. The objectives of this study were to identify keystone bacterial taxa in the seed-associated and phyllosphere microbiomes of *A. inebrians*, determine whether these taxa were represented among culturable bacterial isolates, and evaluate the growth-promoting effects of selected *Pseudomonas* strains.

## MATERIALS AND METHODS

### Study site and experimental design

This study site was located in the College of Pastoral Agriculture Science and Technology, Yuzhong campus of Lanzhou University (104°39′E, 35°89′N; altitude: 1,653 m). Twelve plots (each 4.8 × 4.0 m) were established in 2017. Each plot housed two subplots, which were separated by a 1 m deep concrete wall and planted with either endophyte-infected (EI) or endophyte-free (EF) *A. inebrians* plants.

The experimental plants were derived from a single *A. inebrians* individual that was confirmed to be infected with *E. gansuensis* via microscopic detection of characteristic intercellular hyphae in leaf sheaths stained with aniline blue (2). In 2011, seeds collected from this plant were divided into two batches. One batch was treated with thiophanate-methyl to eliminate the endophyte, thereby producing EF seeds, while the other batch remained untreated to preserve the EI status. Subsequently, in 2012, 200 seedlings from each batch were separately cultivated at the experimental site to establish foundational EI and EF seed stocks. The endophyte status of representative plants from both populations was verified before sampling (43, 44).

### Sampling strategy

*A. inebrians* seeds were collected from eight randomly selected plots. Within each subplot, seeds were obtained from three mature plants and pooled to create a composite sample (44). The third or fourth youngest fully expanded leaves were collected from tillers at five sampling points within each subplot and pooled to form one composite sample. From the pooled leaves, 15–17 cm segments were aseptically excised using sterilized scissors after removing the leaf sheath and tip (43). For rhizosphere soil sampling, five *A. inebrians* plants were randomly selected from each subplot. Soil cores were collected to a depth of 20 cm from the root zones of each selected plant. After the plants and associated roots were removed from the soil cores, loosely attached soil was gently shaken off, and the soil remaining tightly attached to the root surfaces was collected as rhizosphere soil (40). The five rhizosphere soil subsamples from each subplot were pooled to form one composite sample.

All samples were transported to the laboratory in an insulated container with ice packs. Samples used for culture-dependent bacterial isolation were stored at 4°C in a refrigerator, whereas samples used for DNA extraction were stored at −80°C in a refrigerator.

For clarity, treatment abbreviations were defined according to plant compartment, microbial location, and *Epichloë* infection status. SEnI and SEnF denote seed endophytic bacterial communities of *Epichloë*-infected and endophyte-free plants, respectively; SEpI and SEpF denote seed epiphytic bacterial communities; GEpI and GEpF denote glume epiphytic bacterial communities; LEnI and LEnF denote leaf endophytic bacterial communities; LEpI and LEpF denote leaf epiphytic bacterial communities; and REI and REF denote rhizosphere soil bacterial communities of *Epichloë*-infected and endophyte-free plants, respectively.

### Seed and leaf sample amplicon sequencing

A total of 16 seed samples (eight EI and eight EF) were used to detect seed epiphytic and endophytic microbes. In addition, 16 glume samples (eight EI and eight EF) were analyzed for glume epiphytic microbes. Finally, a total of 16 leaf samples (eight EI and eight EF) were used to detect both endophytic and epiphytic microbes. Total DNA was extracted from these seeds and leaf samples using an MN NucleoSpin 96 Soil DNA Kit. The V3-V4 region of the bacterial 16S rRNA genes was amplified using the primer pair 335F (5′-CADACTCCTACGGGAGGC-3′) and 769R (5′-ATCCTGTTTGMTMCCCVCRC-3′). High-throughput sequencing of bacterial 16S rRNA genes was performed on the purified, pooled samples using the Illumina NovaSeq 6000 platform at Biomarker Technologies Corporation (Beijing, China). The raw sequencing data for the seed-borne bacterial communities were originally published in reference 44 and are publicly available under BioProject PRJNA865502. The raw sequencing data for the phyllosphere bacterial communities were originally published in reference 43 and are available under BioProject PRJNA795658. In the present study, these data sets were re-analyzed with a focus on constructing co-occurrence networks to identify keystone taxa, which was not performed in the original publications.

### Network construction with the random matrix theory (RMT) approach

Species interaction networks, also known as phylogenetic molecular ecological networks (pMENs), were constructed based on 16S rRNA sequencing data (28). Unlike other methods, this approach uses RMT to automatically determine the appropriate similarity threshold (*St*) before network construction (46). The network construction process involved four primary steps: data collection, data transformation, pairwise similarity matrix calculation, and adjacency matrix determination (28). All analyses were conducted using the Molecular Ecological Network Analysis Pipeline (http://ieg4.rccc.ou.edu/MENA/) (28). Random networks were generated by rewiring all links in the pMENs while maintaining the same number of links and nodes as in the empirical pMENs.

### Detection of modules and identification of node roles

In this study, a module refers to a group of nodes or operational taxonomic units (OTUs) that are more closely connected within the group than with other groups. Networks were divided into several modules using the greedy modularity optimization method (28). Modularity (*M*) is an index that measures whether a network can be divided into modules (29, 46, 47). The detection of modular topological roles is essential for identifying keystone taxa based on the roles of the nodes within modules. The topological roles were classified using within-module connectivity (*Zi*) and among-module connectivity (*Pi*) values. Nodes were categorized into four groups based on their *Zi* and *Pi* values: peripherals (nodes connected in modules with few outside connections, with *Zi* ≤ 2.5 and *Pi* ≤ 0.6); connectors (nodes that connect modules, with *Zi* ≤ 2.5 and *Pi* > 0.6); module hubs (highly connected nodes within modules, with *Zi* > 2.5 and *Pi* ≤ 0.6); and network hubs (highly connected nodes within the entire network, with *Zi* > 2.5 and *Pi* > 0.6) (28, 47).

### Isolation and identification of cultivable bacteria from *A. inebrians* seed, leaf, and rhizosphere soil

A total of 100 seeds (50 EI and 50 EF), 10 g of fresh leaves (5 g EI and 5 g EF), and 10 g of fresh rhizosphere soil (5 g EI and 5 g EF) from *A. inebrians* were suspended in PBS buffer. Detailed protocols for seed epiphytic, seed endophytic, and glume epiphytic treatments are described in Liang et al. (44), whereas treatments for leaf epiphytic and endophytic bacteria are provided in reference 45. The samples were diluted to different concentrations (10^−1^–10^−7^) and plated on R_2_A agar medium, which contained yeast extract (0.5 g/L), proteose peptone (0.5 g/L), casein hydrolysate (0.5 g/L), glucose (0.5 g/L), soluble starch (0.5 g/L), sodium pyruvate (0.3 g/L), dipotassium hydrogen phosphate (0.3 g/L), magnesium sulfate anhydrous (0.024 g/L), and agar (15.0 g/L), adjusted to pH 7.2 ± 0.2. After 3–7 days of incubation, bacterial colonies were purified based on their morphological characteristics. The nearly full-length bacterial 16S rRNA gene was amplified using primers 27F (5′-AGAGTTTGATCCTGGCTC-3′) and 1492R (5′-CGGCTACCTTGTTACGACTT-3′). PCR products were verified by agarose gel electrophoresis and sequenced by Sanger sequencing at Sangon Biotech (Shanghai, China).

### Alpha and beta diversity analyses

Alpha diversity was assessed using the Shannon (https://mothur.org/wiki/shannon/), Simpson (https://mothur.org/wiki/simpson/), Chao (https://mothur.org/wiki/chao/), and ACE indices (https://mothur.org/wiki/ace/) using Mothur software (v1.30).

Beta diversity was analyzed using the Bray–Curtis distance matrix, followed by principal coordinate analysis. Permutational multivariate analysis of variance (PERMANOVA) was conducted to evaluate differences in community structure using the “vegan” package in R (v4.0.3).

### *Pseudomonas* strains were selected based on the differences in the levels of 16S rRNA

To select *Pseudomonas* strains for functional validation, we first performed preliminary identification of culturable isolates based on their 16S rRNA gene sequences. For each ecological niche, we constructed a phylogenetic tree using all obtained *Pseudomonas* strains. Strains falling into distinct phylogenetic clades were considered to represent different species-level groups. From each niche, two representative strains from different groups were selected for further research.

### Physiological and biochemical responses of *A. inebrians* to *Pseudomonas* inoculation

To investigate the effects of *Pseudomonas* inoculation on the growth and development of *A. inebrians* under sterile conditions, we conducted a 45-day pot experiment using sterilized vermiculite mixed (2:1, vol/vol) with commercially available black potting soil (treated at 120°C for 5 h in an autoclave). EI and EF seeds were randomly selected, surface-sterilized with 75% alcohol for 2 min, treated with a 1% sodium hypochlorite solution for 5 min, and finally washed four to five times with sterile distilled water. Three EF or EI seeds were sown in each pot, with a total of 28 EF and 28 EI treatment pots grown in a controlled environment greenhouse at Lanzhou University (temperature: 25 ± 2°C; humidity: 46% ± 2%). After the emergence of the second true leaf, each seedling received 50 mL of half-strength Hoagland nutrient solution every 2 days (45). The nutrient solution was prepared and contained 2.975 mM KNO_3_, 2.0 mM Ca(NO_3_)_2_·4H_2_O, 1.0 mM NH_4_H_2_PO_4_, 0.5 mM MgSO_4_·7H_2_O, 25 μM KCl, 12.5 μM H_3_BO_3_, 1.0 μM MnSO_4_·H_2_O, 1.0 μM ZnSO_4_·7H_2_O, 0.25 μM CuSO_4_·5H_2_O, 0.25 μM H_2_MoO_4_, and 25 μM Fe supplied as NaFeDTPA. One-month-old EI and EF seedlings were inoculated in the root zone with 10 mL of a *Pseudomonas* culture grown in Luria–Bertani (LB) liquid medium and adjusted to an OD_600_ of 0.9. The LB medium contained 5 g/L yeast powder, 5 g/L NaCl, and 10 g/L tryptone. Control plants received 10 mL of sterile LB medium without bacteria. Hereafter, this treatment is referred to as the control. Inoculation was repeated at 1 and 2 weeks after the initial application to promote *Pseudomonas* colonization. After inoculation, the pots were regularly randomized, and water was added daily to maintain saturated soil moisture conditions.

#### Fresh and dry biomass measurement

Forty-five days after bacterial inoculation, *A. inebrians* plants were removed from the pots, and the roots were washed to remove the potting mixture while maintaining root integrity. After washing, the plants were dried on absorbent paper, and the roots were excised. The fresh weight of each plant was recorded from two parts, and the average fresh weight of all plants under each treatment was calculated. The plant samples were then placed in an oven at 40°C for 48 h for dry weight determination.

#### Determination of nutrient content in leaves

Total nitrogen (TN) and total phosphorus (TP) contents in the leaves were measured using a fully automated flow injection analyzer (FIAstar 5000 Analyzer, FOSS Analytical, Denmark) (48). Total potassium (TK) content of the leaves was determined using flame photometry following the method described in reference 49. Crude protein, crude fiber, and ether extract content were analyzed using the Kjeldahl method (50), the Van Soest method (51), and the Soxhlet extraction method (52), respectively.

### Whole-genome sequencing, assembly, and taxonomic assignment of *Pseudomonas*

*Pseudomonas* strains were cultured using LB liquid medium and incubated at 28 ± 2°C with shaking at 180 rpm for 48 h. A total of 1.5 mL volume of bacterial solution was transferred to a 2 mL aseptic centrifuge tube, centrifuged at room temperature at 14,000 × *g* for 1 min, followed by discarding all the medium. The bacterial precipitation was quickly frozen in liquid nitrogen for 3–4 h, then transferred to −80°C for preservation, and then transported with dry ice to Sangon Biotech (Shanghai, China) for bacterial whole-genome sequencing.

Paired-end libraries were prepared and sequenced on an Illumina NovaSeq 6000 platform, and long-read sequencing was performed on a PacBio RS II System at Sangon Biotech (Shanghai, China) (53). Raw reads were quality-trimmed using Trimmomatic v0.30 with a sliding window quality cutoff of Q15. *De novo* hybrid assembly was performed using Unicycler v0.4.8. Genome completeness and contamination were assessed using BUSCO v5.2.2 (54). Species assignment was determined by calculating the average nucleotide identity (ANI) using the OrthoANIu algorithm (threshold >95% for same species).

Whole-genome sequences of six type strains of *Pseudomonas* species were included as taxonomic references. The genome assemblies of *P. cucumis* FP1935^T^ (GenBank assembly accession GCA_030687935.1) and *P. atacamensis* M7D1^T^ (GCA_004801935.1) were used for ANI-based species assignment of the isolates. Four additional type-strain genomes, *P. putida* NBRC 14164^T^ (GCA_000412675.1), *P. fluorescens* ATCC 13525^T^ (GCA_900215245.1), *P. synxantha* DSM 18928^T^ (GCA_001439725.1), and *P. chlororaphis* subsp. *chlororaphis* DSM 50083^T^ (GCA_003945765.1) were included to provide broader taxonomic context within the genus. Pairwise average nucleotide identity values were calculated using the OrthoANIu algorithm. An ANI similarity matrix was generated, and hierarchical clustering was performed based on the pairwise ANI values to visualize genomic relatedness among the isolates and reference genomes. Genome circular maps were generated using Circos software (55).

### Statistical analysis

Analyses of culturable bacterial community composition and genus-level abundance (e.g., the relative abundance of *Pseudomonas*) were based on the relative proportions of bacterial isolates obtained from colony counts for each sample. Two-way analysis of variance (ANOVA) was used to evaluate the main effects of *Epichloë* infection status, plant compartment, and their interaction on the alpha-diversity indices of culturable bacterial communities. The same model was applied to analyze the relative abundance and the number of *Pseudomonas* isolates across the seed, leaf, and rhizosphere compartments.

For the plant inoculation experiment, two-way ANOVA was used to evaluate the main effects of *Epichloë* infection status, *Pseudomonas* inoculation treatment, and their interaction on plant length, branch number, total nitrogen, total phosphorus, total potassium, crude protein, crude fiber, and ether extract. Within each endophyte infection status (EI or EF), one-way ANOVA was used to compare plant fresh weight and dry weight among *Pseudomonas* inoculation treatments. When ANOVA indicated significant effects, mean values were compared using Duncan’s multiple-range test (*P* < 0.05). Independent-samples *t*-tests were used only for specified pairwise comparisons between EI and EF *A. inebrians* plants. Differences were considered statistically significant at *P* < 0.05.

The network construction and statistical analysis were performed using the existing pipeline available at Molecular Ecological Network Analysis Pipeline (http://ieg4.rccc.ou.edu/MENA/) (28). All visual modules in these networks were graphed according to the Gephi (v.0.9.5) software. Additional statistical analyses and figure generation in this study were carried out using R (v.3.6.3) and Adobe Illustrator (v.2024).

## RESULTS

### *Epichloë* endophyte shapes microbial networks in *A. inebrians*

**Fig 1:**
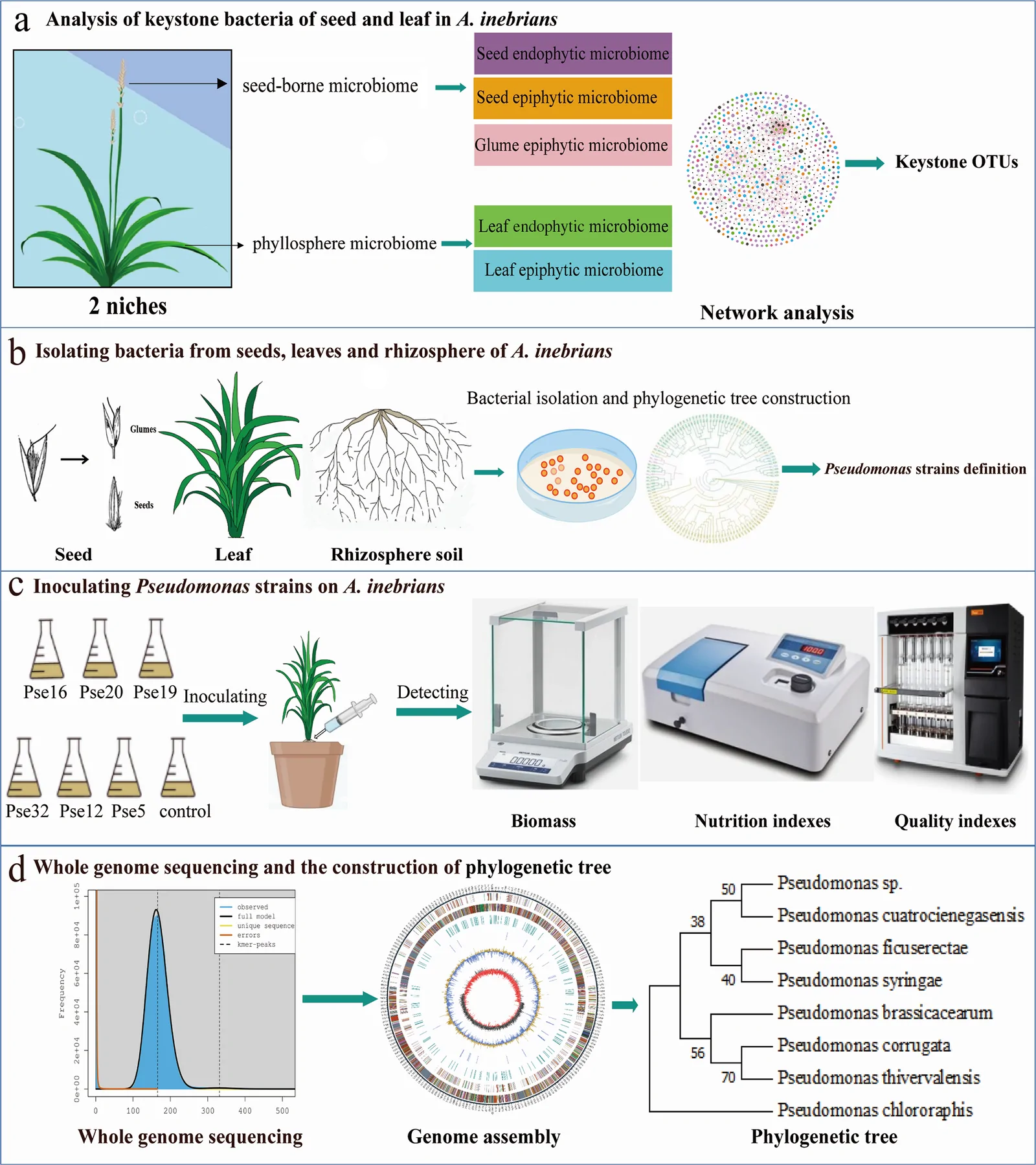
Overview of the experimental workflow for this study. (**a**) Analysis of keystone species of seed and leaf bacteria in *Achnatherum inebrians* using the phylogenetic molecular ecological network analysis method. (**b**) Isolation of bacteria from seed, leaf, and rhizosphere of *A. inebrians*. (**c**) Inoculation of *Pseudomonas* strains on *A. inebrians*. (**d**) Whole-genome sequencing and the construction of phylogenetic tree of six *Pseudomonas* strains.

**Fig 2:**
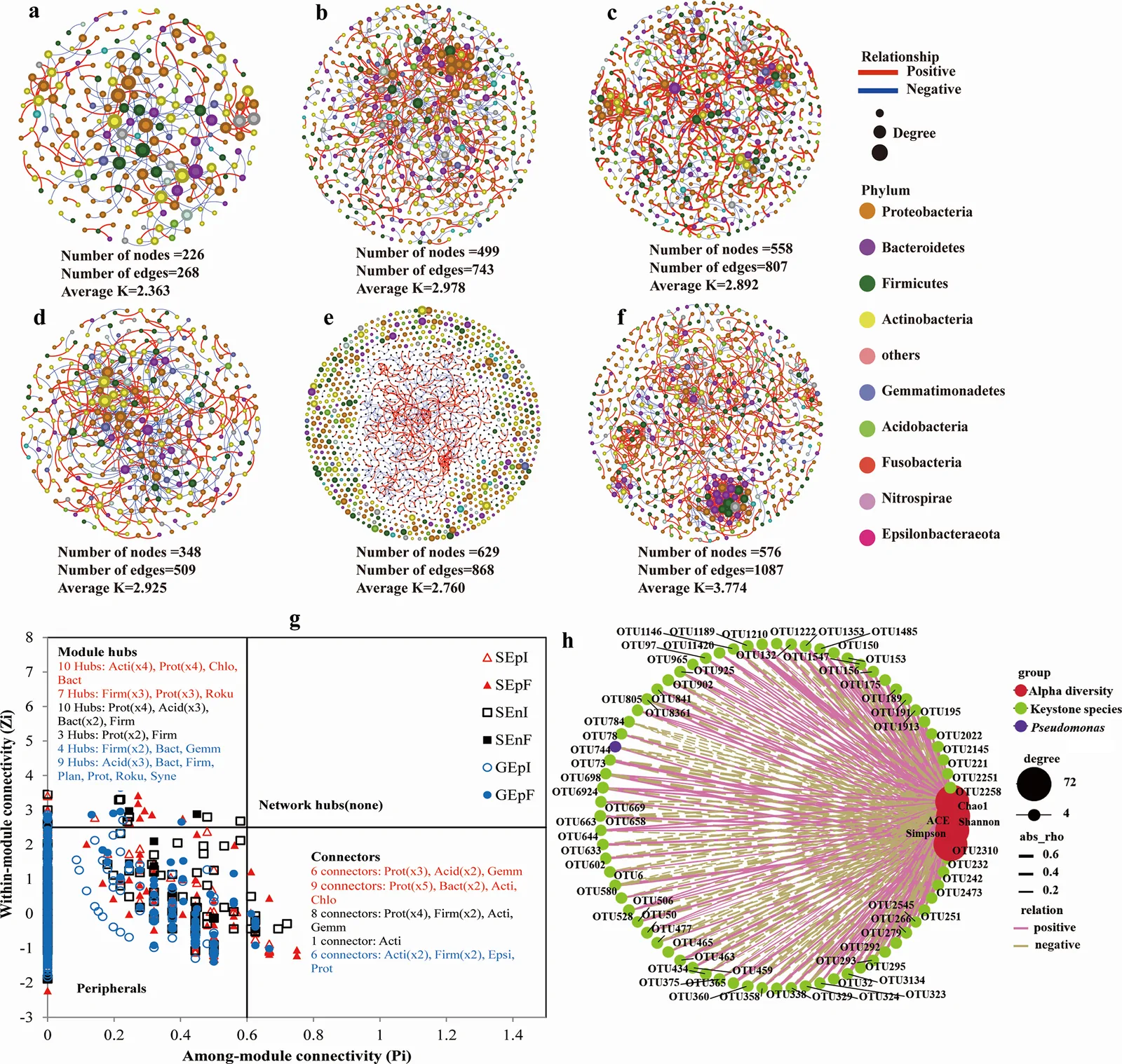
Network structure analysis of seed-borne bacterial communities. (**a**) Seed endophytic microbes of endophyte-free (EF) *A. inebrians* (SEnF). (**b**) Seed epiphytic microbes of EF *A. inebrians* (SEpF). (**c**) Glume epiphytic microbes of EF *A. inebrians* (GEpF). (**d**) Seed endophytic microbes of endophyte-infected (EI) *A. inebrians* (SEnI). (**e**) Seed epiphytic microbes of EI *A. inebrians* (SEpI). (**f**) Glume epiphytic microbes of EI *A. inebrians* (GEpI). (**g**) The topological roles of nodes in bacterial networks from SEpI, SEpF, SEnI, SEnF, GEpI, and GEpF are illustrated in a *Z*–*P* plot. Module hubs have within-module connectivity (*Zi*) > 2.5 and among-module connectivity (*Pi*) < 0.6, whereas connectors have *Zi* < 2.5 and *Pi* > 0.6. The bacterial phyla of the hubs and connectors are listed on the plot using the following abbreviations: Prot, Proteobacteria; Acti, Actinobacteria; Firm, Firmicutes; Bact, Bacteroidetes; Acid, Acidobacteria; Gemm, Gemmatimonadetes; Chlo, Chloroflexi; Roku, Rokubacteria; Epsi, Epsilonbacteraeota; Plan, Planctomycetales; and Syne, Synergistetes. (**h**) Spearman’s correlation networks between seed-associated bacterial α-diversity indices and seed-associated keystone taxa at the genus level in *A. inebrians*.

**Fig 3:**
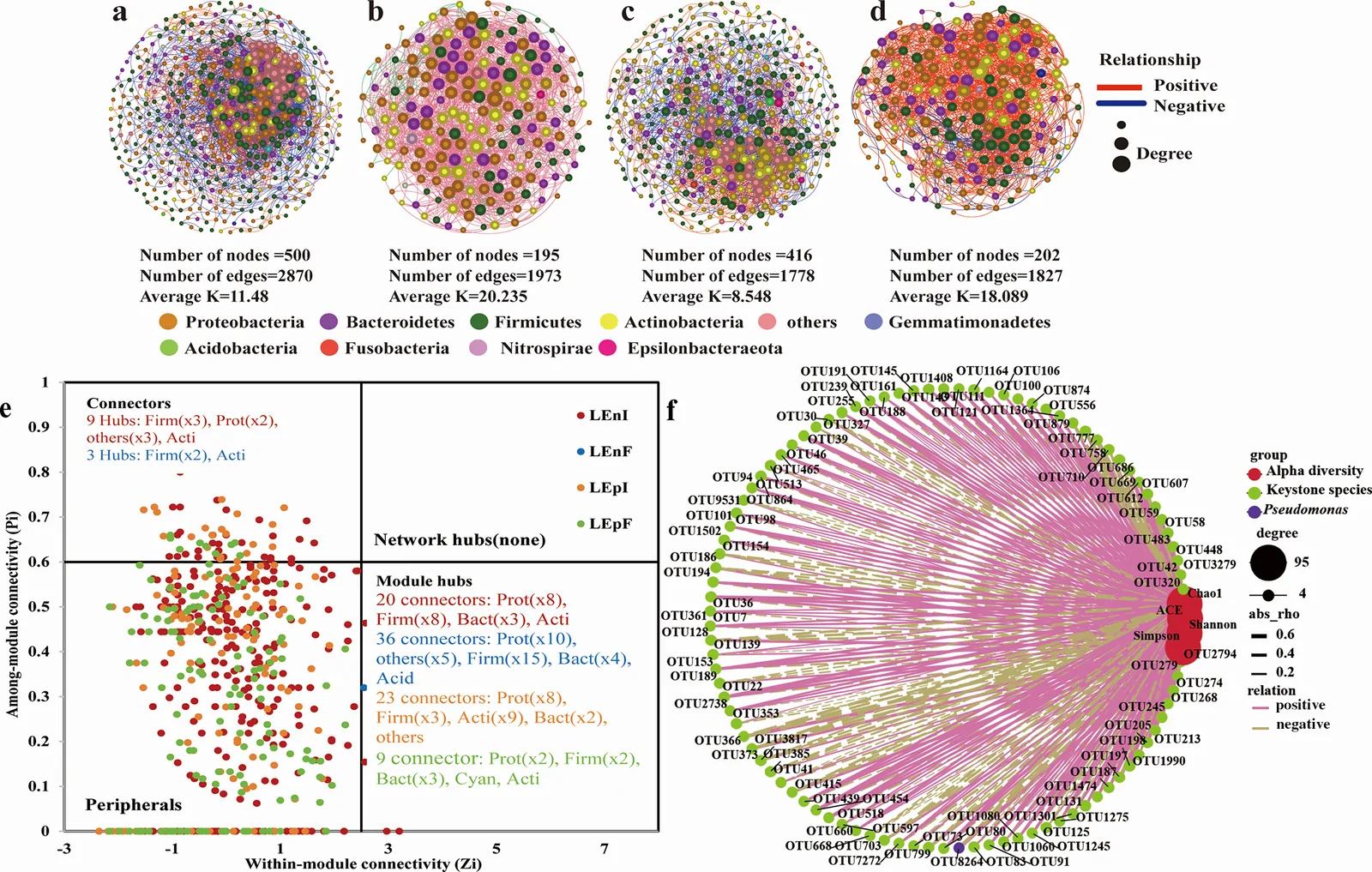
Network structure analysis of phyllosphere bacterial communities. (**a**) Leaf epiphytic microbes of EF *A. inebrians* (LEpF). (**b**) Leaf endophytic microbes of EF *A. inebrians* (LEnF). (**c**) Leaf epiphytic microbes of EI *A. inebrians* (LEpI). (**d**) Leaf endophytic microbes of EI *A. inebrians* (LEnI). (**e**) The topological roles of nodes in bacterial networks from LEpI, LEpF, LEnI, and LEnF are illustrated in the *Z–P* plot. (**f**) Spearman’s correlation networks between phyllosphere bacterial α-diversity indices and phyllosphere keystone taxa at the genus level in *A. inebrians*.

To understand microbial interactions within EI and EF *A. inebrians*, we constructed bacterial co-occurrence networks for both seed-borne and phyllosphere communities using an RMT-based approach (Fig. 1a). The empirical pMENs differed from the corresponding random networks. All empirical pMENs exhibited scale-free and small-world characteristics, with short average geodesic distances ranging from 2.6 to 9.2. Network complexity varied among plant compartments and *Epichloë* infection treatments. *Epichloë* infection increased average connectivity (Avg K) in the seed endophytic and glume epiphytic networks but reduced Avg K in the seed epiphytic, leaf epiphytic, and leaf endophytic networks (Tables 1 and 2). The seed-borne bacterial networks were highly interconnected. In EF plants, the seed epiphytic network exhibited greater complexity than the seed endophytic and glume epiphytic networks. In contrast, in EI plants, the glume epiphytic network exhibited the highest complexity among seed-associated compartments (Fig. 2; Table 1). In the phyllosphere, *Epichloë* infection reduced bacterial network complexity in both leaf epiphytic and leaf endophytic compartments. Nevertheless, the leaf endophytic network remained more complex than the leaf epiphytic network (Fig. 3; Table 2). Overall, the phyllosphere networks exhibited higher Avg K values than the seed-borne networks.

**TABLE 1:** Topological properties of the empirical phylogenetic molecular ecological networks (pMENs) of bacterial communities under SEpF, SEpI, SEnF, SEnI, GEpF, GEpI, and their associated random pMENs^*a*^

Treatment	Empirical networks							Random networks		
	Nodes	Links	*R*^2^ of power law	Avg K	Avg geodesic distance	Avg clustering coefficient	Modularity (no. of modules)	Avg geodesic distance ± SD	Avg clustering coefficient ± SD	Modularity ± SD
SEnF	226	268	0.857	2.363	9.159	0.117	0.864 (31)	6.038 ± 0.204	0.007 ± 0.005	0.725 ± 0.009
SEnI	348	509	0.856	2.925	5.762	0.096	0.739 (36)	4.833 ± 0.076	0.011 ± 0.004	0.630 ± 0.007
SEpF	499	743	0.901	2.978	8.100	0164	0.844 (56)	5.176 ± 0.051	0.007 ± 0.003	0.634 ± 0.006
SEpI	629	868	0.864	2.760	9.548	0.139	0.878 (72)	5.867 ± 0.076	0.004 ± 0.002	0.679 ± 0.006
GEpF	558	807	0.863	2.892	8.377	0.127	0.869 (60)	5.452 ± 0.067	0.006 ± 0.003	0.653 ± 0.006
GEpI	576	1,087	0.897	3.774	8.898	0.240	0.871 (66)	4.410 ± 0.045	0.012 ± 0.003	0.530 ± 0.005

^*a*^ Random networks: 100 networks generated by rewiring all of the links of a pMEN with the identical numbers of nodes and links to the corresponding empirical pMEN. Treatments: seed epiphytic microbes of endophyte-infected *A. inebrians *(SEpI), seed epiphytic microbes of endophyte-free *A. inebrians *(SEpF), seed endophytic microbes of endophyte-infected *A. inebrians *(SEnI), seed endophytic microbes of endophyte-free *A. inebrians *(SEnF), glume microbial community of endophyte-infected *A. inebrians *(GEpI), and glume microbial community of endophyte-free *A. inebrians *(GEpF) network.

**TABLE 2:** Topological properties of the empirical phylogenetic molecular ecological network of bacterial communities under LEpF, LEpI, LEnF, and LEnI, and their associated random pMENs^*a*^

Treatment	Empirical networks							Random networks		
	Nodes	Links	*R*^2^ of power law	Avg K	Avg geodesic distance	Avg clustering coefficient	Modularity (no. of modules)	Avg geodesic distance ± SD	Avg clustering coefficient ± SD	Modularity ± SD
LEpF	500	2,870	0.91	11.48	4.197	0.255	0.422	2.96 ± 0.019	0.007 ± 0.003	0.226 ± 0.004
LEpI	416	1,778	0.816	8.548	4.333	0.289	0.546	3.098 ± 0.021	0.004 ± 0.003	0.29 ± 0.005
LEnF	195	1,973	0.0079	20.235	2.675	0.494	0.409	2.151 ± 0.01	0.015 ± 0.007	0.161 ± 0.005
LEnI	202	1,827	0.108	18.089	2.930	0.452	0.43	2.244 ± 0.015	0.012 ± 0.006	0.172 ± 0.005

^*a*^ Random networks: 100 networks generated by rewiring all of the links of a pMEN with the identical numbers of nodes and links to the corresponding empirical pMEN. Treatments: leaf epiphytic microbes of endophyte-infected *A. inebrians *(LEpI), leaf epiphytic microbes of endophyte-free *A. inebrians *(LEpF), leaf endophytic microbes of endophyte-infected *A. inebrians *(LEnI), and leaf endophytic microbes of endophyte-free *A. inebrians *(LEnF).

Keystone species analysis identified *Pseudomonas* as a dominant genus in both seed-borne and phyllosphere bacterial communities (Tables S1 and S2), indicating its crucial ecological role in maintaining microbiome stability. At the phylum level, Proteobacteria and Firmicutes were the most influential microbial groups, forming the structural backbone of these bacterial networks (Fig. 2g and 3e; Tables S1 and S2). Several other genera, including *Lachnospiraceae*, *Sphingomonas*, and *Actinoplanes*, also played significant roles, although their influence varied across plant compartments (Tables S1 and S2). Seed-associated keystone species showed 114 significant (*P* < 0.05) correlations with alpha diversity; characteristically, OTU744 was significantly (*P* < 0.05) positively correlated with bacterial community Chao1, ACE, and Simpson diversities (Fig. 2h; Table S3). A total of 19 significant (*P* < 0.05) correlations between phyllosphere keystone species and alpha diversity, especially OTU8264, significantly (*P* < 0.05) negatively correlated with bacterial community Chao1 and ACE diversity indices (Fig. 3f; Table S4). The distinct network structures observed between seed-borne and phyllosphere microbiomes highlight the niche-specific assembly of microbial communities, with *Epichloë* endophyte infection altering both the overall complexity and microbial interactions within these networks.

Beyond shaping microbial composition, *Epichloë* endophyte presence had significant implications for plant–microbe interactions. The reduced network complexity in EI plants may indicate a shift toward a more specialized and stable microbial consortium, potentially optimizing plant health and nutrient acquisition. After establishing that the *Epichloë* endophyte reshapes bacterial network architecture and identifies *Pseudomonas* as a keystone genus (Tables S1 and S2), we aimed to determine whether these *in silico* predictions corresponded to culturable populations.

### Culturable bacterial community assembly and diversity across seed, leaf, and rhizosphere compartments of *A. inebrians*

**Fig 4:**
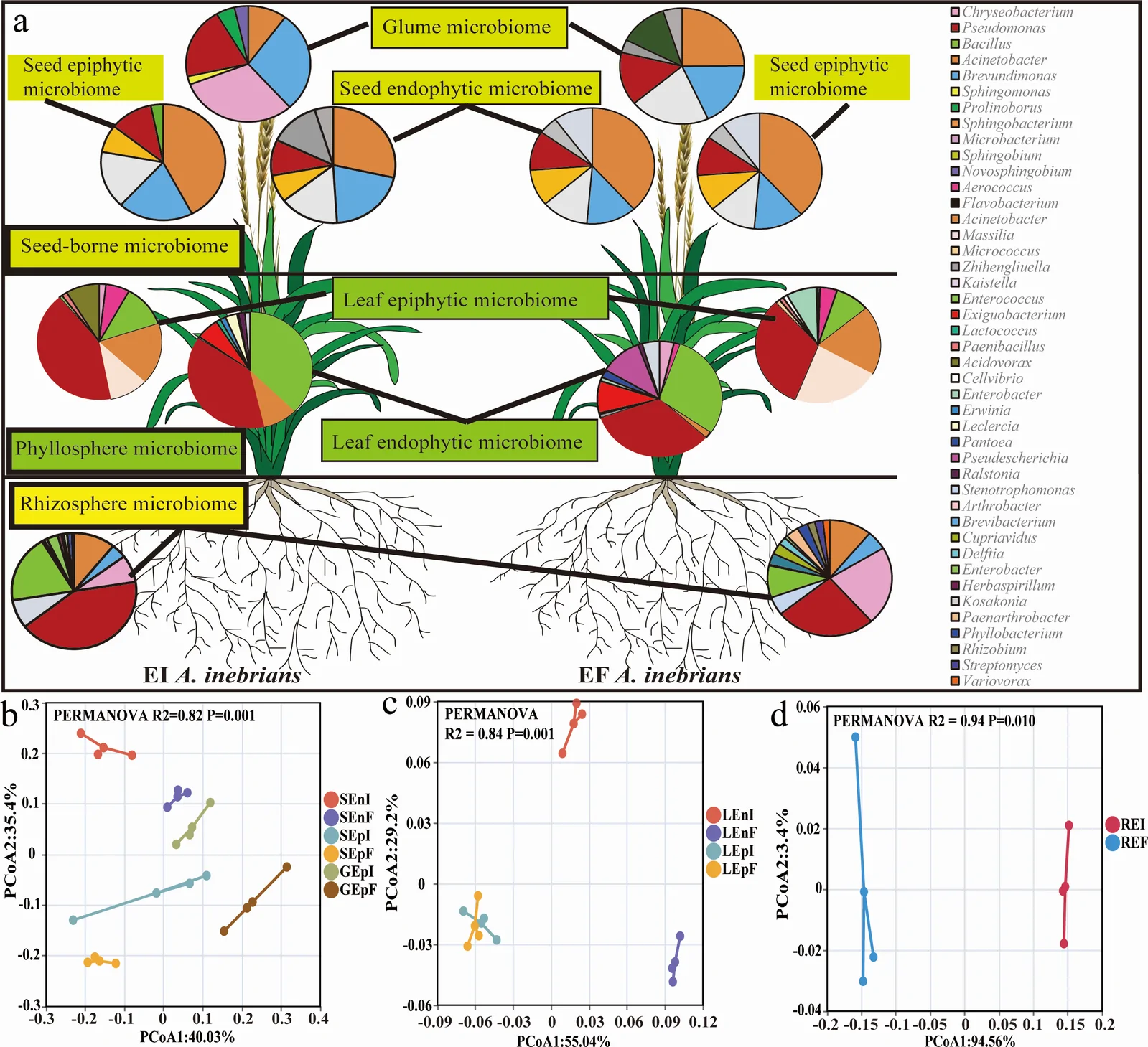
Composition and beta-diversity of culturable bacterial communities isolated from *A. inebrians*. (**a**) Relative abundance of dominant genera. (**b–d**) Principal coordinate analysis (PCoA) of bacterial communities from seed (**b**), leaf (**c**), and rhizosphere (**d**) compartments based on Bray–Curtis dissimilarity. REI, rhizosphere microbes of EI *A. inebrians*; REF, rhizosphere microbes of EF *A. inebrians*.

To investigate potential links between keystone species and host development, we isolated and characterized 1,200 bacterial strains from the seed (epiphytic, endophytic, and glume), leaf (epiphytic and endophytic), and rhizosphere compartments of *A. inebrians* (Fig. 1b). Taxonomic profiling revealed seven dominant genera, *Pseudomonas*, *Bacillus*, *Sphingomonas*, *Acinetobacter*, *Chryseobacterium*, *Brevundimonas*, and *Massilia*, each exhibiting compartment-specific colonization patterns (Fig. 4a; Tables S5 to S7).

The seed compartments (565 isolates) exhibited significant differences in bacterial composition (PERMANOVA, *P* < 0.05) (Fig. 4b; Table S5). Notably, the relative abundance of *Acinetobacter* was significantly (*P* < 0.05) higher than that in the other compartments. SEnI exhibited the highest relative abundance of *Pseudomonas, Brevundimonas*, and *Chryseobacterium*, whereas SEpF and GEpF had the highest relative abundance of *Sphingomonas* (Table S8). Leaf compartments (412 isolates) displayed significant differences in bacterial composition (PERMANOVA, *P* < 0.05) (Fig. 4c; Table S6). The relative abundance of *Chryseobacterium*, *Aerococcus*, *Bacillus*, *Pseudomonas*, *Micrococcus*, *Kaistella*, *Lactococcus*, *Acidovorax*, *Cellvibrio*, and *Stenotrophomonas* varied significantly (*P* < 0.05) between leaf epiphytic and endophytic compartments of EI and EF *A. inebrians* (Table S9). Specifically, LEnI, LEpI, and LEpF exhibited significantly higher relative abundance of *Pseudomonas* (*P* < 0.05) than LEnF, whereas LEnI had the highest relative abundance of *Bacillus* (Table S9). Rhizosphere communities (247 isolates) displayed no significant differences in bacterial composition (PERMANOVA, *P* > 0.05) (Fig. 4d; Table S7), with *Pseudomonas* (EI, 41.33%; EF, 26.33%) and *Bacillus* (EI, 19.35%; EF, 9.35%) dominating. However, *Epichloë* endophyte infection significantly altered the relative abundance of keystone taxa (*P* < 0.05) (Table S10). Particularly, EI had a significantly higher relative abundance of *Pseudomonas* and *Bacillus* than EF *A. inebrians* (Table S10).

**Fig 5:**
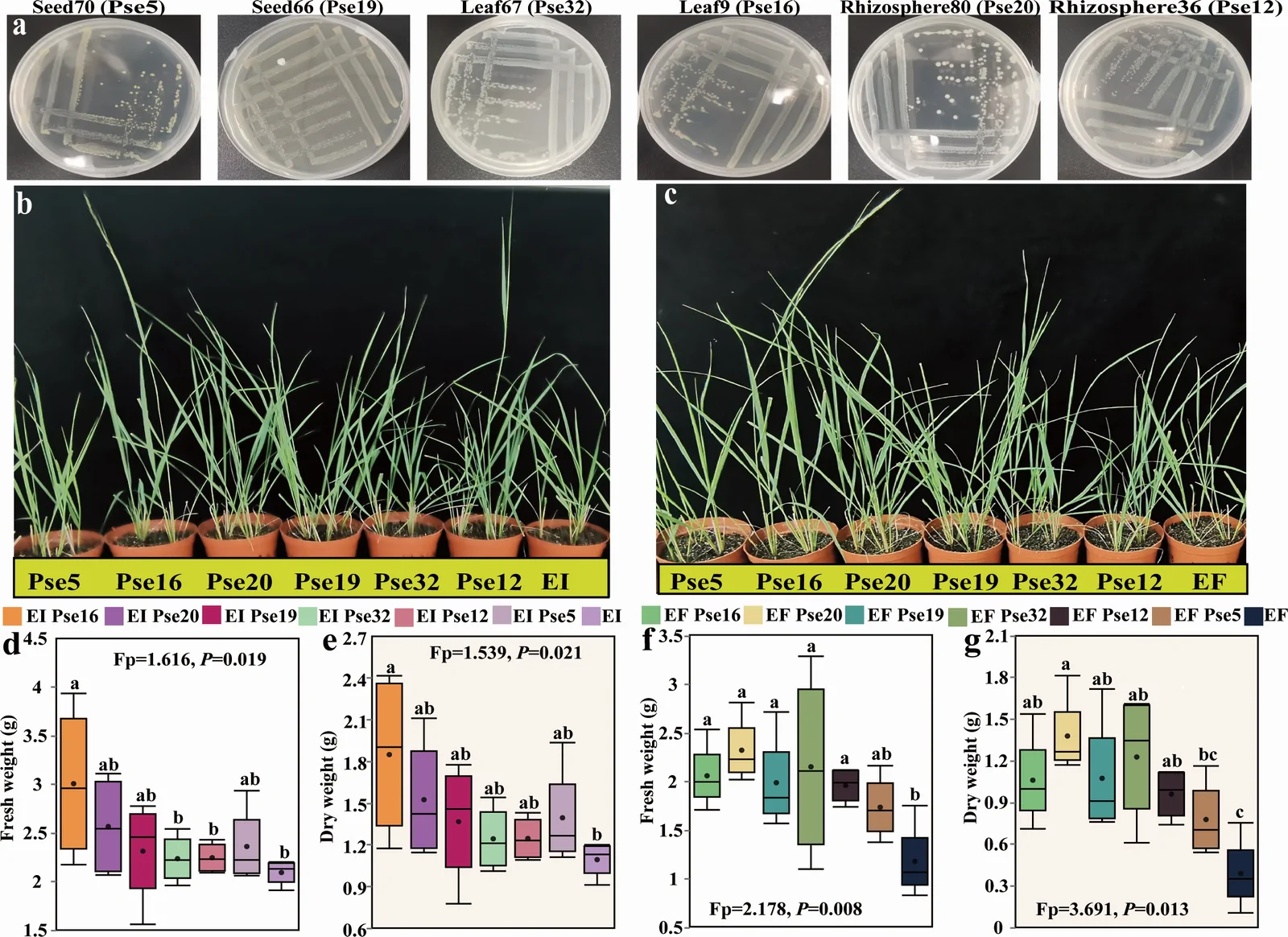
Plant growth-promoting effects of six *Pseudomonas* strains. (**a**) Colony morphology of the six *Pseudomonas* strains on R_2_A plates. (**b and c**) Phenotypic effects of inoculating the six *Pseudomonas* strains on EI *A. inebrians* and EF *A. inebrians*. (**d–g**) Fresh and dry biomass of EI (**d, f**) and EF (**e, g**) plants after inoculation with the six *Pseudomonas* strains. EI: *Epichloë*-infected plants treated with 10 mL of sterile LB medium without bacteria; EF: *Epichloë*-free plants treated with 10 mL of sterile LB medium without bacteria; Pse16, Pse20, Pse19, Pse32, Pse12, and Pse5 represent inoculation with different *Pseudomonas* strains; E: *Epichloë* infection status; and P: *Pseudomonas* inoculation in EI and EF *A. inebrians*. Different lowercase letters indicate significant differences (*P* < 0.05) among different treatments. Asterisks (*) represent significant differences between EI and EF.

We assessed the overall impact of the *Epichloë* endophyte on the culturable bacterial communities. Alpha diversity analysis revealed compartment-specific patterns (Fig. S1a through c), with endophytic communities generally exhibiting higher diversity than epiphytic communities. Notably, principal coordinate analysis (PCoA) showed that the endophyte infection status significantly (*P* < 0.05) altered the bacterial diversity and community structure, particularly in the seed and leaf compartments (Fig. 4b through d). These findings confirm that the *Epichloë* endophyte reshapes the microbial habitat of the host grass. Within this reconfigured bacterial community, we determined that the genus *Pseudomonas* was dominant and exhibited significant variation in its abundance across compartments and endophyte status (Fig. 5d), therefore prompting further investigation into its ecological role. The high abundance and niche-specific distribution of culturable *Pseudomonas* prompted us to functionally test whether these isolates, particularly those phylogenetically linked to the keystone OTUs, could directly influence host plant performance.

### Abundance, distribution, and selection of representative *Pseudomonas* strains across compartments

Next, we focused on the *Pseudomonas* genus across seed, leaf, and rhizosphere compartments. The results indicated that the *Epichloë* infection status, plant compartment, and their interaction significantly influenced *Pseudomonas* abundance (*P* < 0.05) (Fig. S1d) and the number of *Pseudomonas* strains (Fig. S1e). The rhizosphere exhibited the highest *Pseudomonas* abundance and strain count compared to the phyllosphere and seed-associated compartments. Notably, the phyllosphere displayed higher *Pseudomonas* abundance and strain counts than the seed-associated compartment. REI had significantly higher *Pseudomonas* abundance (*P* < 0.05) than REF. LEnF exhibited significantly lower *Pseudomonas* abundance (*P* < 0.05) than LEnI, LEpI, and LEpF. SEnI had significantly higher *Pseudomonas* abundance (*P* < 0.05) than other seed treatments, whereas GEpF exhibited significantly lower *Pseudomonas* abundance (*P* < 0.05) than other treatments (Fig. S1d). Similarly, REI displayed the highest *Pseudomonas* strain count among all treatments, whereas GEpF exhibited the lowest (Fig. S1e).

A complete list of the 273 *Pseudomonas* isolates, including species identification and isolation locations, is provided in Table S11. Based on 16S rRNA preliminary identification, we selected six representative *Pseudomonas* strains from three ecological niches (seed, leaf, and rhizosphere), with two strains from each niche from distinct phylogenetic clades (Fig. 1b). These included Seed70 and Seed66 from seed, leaf9 and leaf67 from leaf, and Rhizosphere80 and Rhizosphere36 from rhizosphere (Fig. S2). For consistency across the study, these strains were renamed as Pse5 (Seed70), Pse19 (Seed66), Pse32 (leaf67), Pse16 (leaf9), Pse20 (Rhizosphere80), and Pse12 (Rhizosphere36), respectively (Fig. 5a).

### Host growth promotion by selected *Pseudomonas* strains

**Fig 6:**
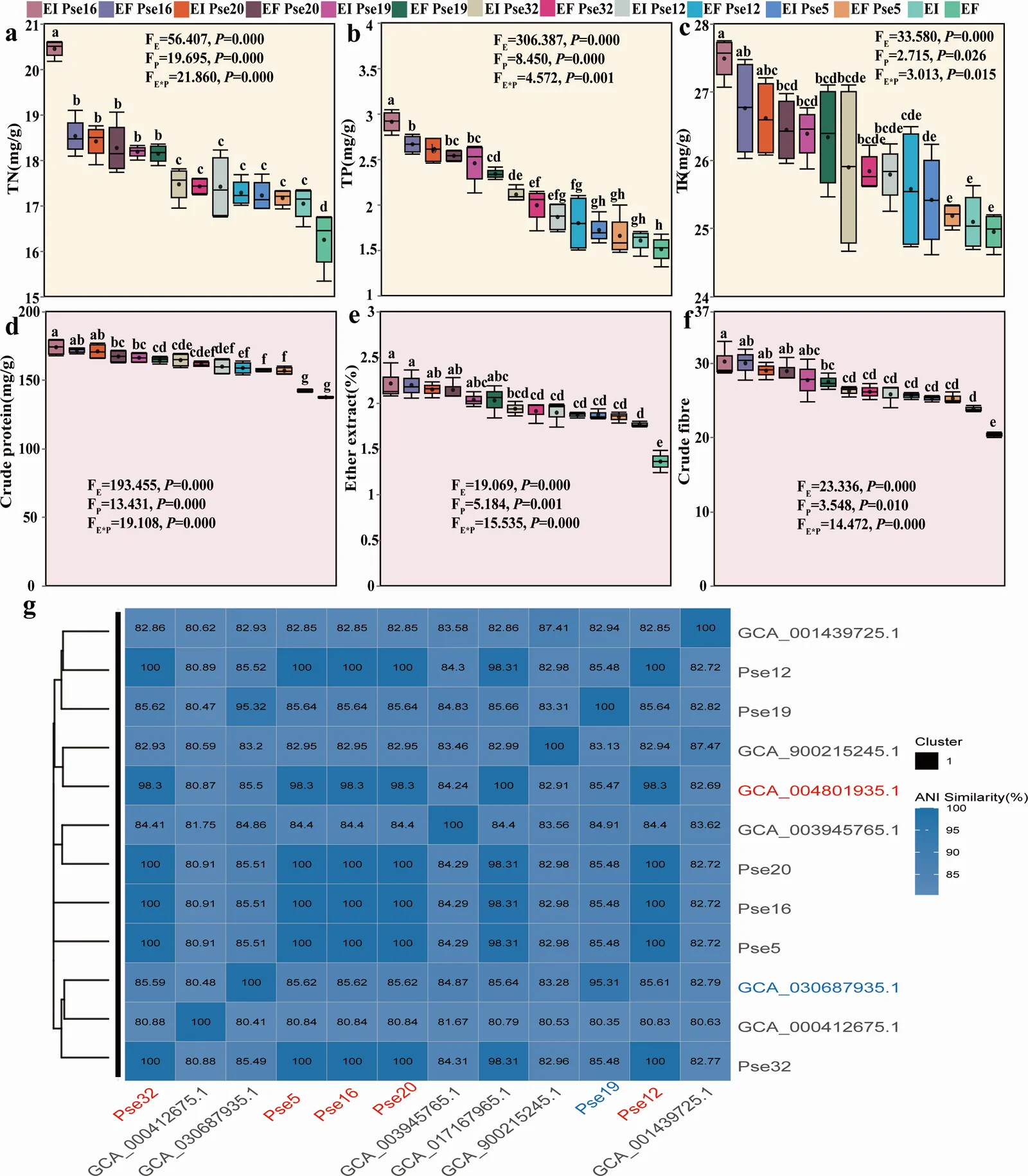
Effects of inoculating six *Pseudomonas* strains on nutritional and quality indices of EI and EF *A. inebrians*. (**a**) Total nitrogen (TN) concentration. (**b**) Total phosphorus (TP) concentration. (**c**) Total potassium (TK) concentration. (**d**) Crude protein content. (**e**) Ether extract content. (**f**) Crude fiber content. E: *Epichloë* infection status; P: *Pseudomonas* inoculation in EI and EF *A. inebrians*. (**g**) The distance heatmap shows the ANI similarity among *Pseudomonas* strains and Pse16, Pse20, Pse19, Pse32, Pse12, and Pse5. The higher the similarity, the darker the color. Note: *P. cucumis* FP1935^T^ (GenBank assembly accession GCA_030687935.1), *P. atacamensis* M7D1^T^ (GCA_004801935.1), *P. putida* NBRC 14164^T^ (GCA_000412675.1), *P. fluorescens* ATCC 13525^T^ (GCA_900215245.1), *P. Synxantha* DSM 18928^T^ (GCA_001439725.1), and *P. chlororaphis* subsp. *chlororaphis* DSM 50083^T^ (GCA_003945765.1).

To assess the effects of selected *Pseudomonas* strains on host growth, we inoculated six candidate *Pseudomonas* strains for inoculation in both EI and EF *A. inebrians* (Fig. 1c). Compared to uninoculated controls, all six strains exhibited growth-promoting effects on *A. inebrians* (Fig. 5b and c). Compared with the control, inoculation with all six *Pseudomonas* strains increased the fresh and dry weight of *A. inebrians*. Among the tested strains, Pse16 produced the greatest biomass-promotion effect and significantly (*P* < 0.05) increased both fresh and dry weights of in EI and EF plants (Fig. 5d through g). Compared to the control, *Pseudomonas* inoculation increased the plant length in *A. inebrians* (Fig. S3a). In addition, *Epichloë* endophyte infection significantly influenced branch number (*P* < 0.05), with EI plants exhibiting a significantly higher branch count than EF plants (Fig. S3b). Strain-specific nutrient acquisition was observed. N/K: Pse16, Pse19, and Pse20 significantly increased the TN and TK in EI plants (*P* < 0.05; Fig. 6a and c). Phosphorus: Pse16, Pse19, Pse20, and Pse32 enhanced P uptake, particularly in EF rhizospheres (*P* < 0.05; Fig. 6b). Notably, all six strains significantly improved forage quality metrics in EI plants (*P* < 0.05). Compared to the control, inoculation with Pse16, Pse20, and Pse19 significantly increased TN and TK content of *A. inebrians* (*P* < 0.05; Fig. 6a and c), whereas Pse16, Pse20, Pse19, and Pse32 significantly increased TP content (*P* < 0.05; Fig. 6b).

### Whole-genome sequencing and phylogenetic relationship analysis

To understand the complete genomes and accurate taxonomic status of the six strains, we conducted whole-genome sequencing and constructed a phylogenetic tree for these strains (Fig. 1d). ANI analyses showed that Pse5, Pse12, Pse16, Pse20, and Pse32 belonged to *Pseudomonas atacamensis*, each showing 98.3% ANI with the type strain genome of *P. atacamensis* M7D1^T^ (GCA_004801935.1). Pse19 was assigned to *Pseudomonas cucumis*, showing an ANI value above 95% with the type-strain genome of *P. cucumis* FP1935^T^ (GCA_030687935.1) (Fig. 6g). The core-genome phylogenetic tree (Fig. S4) clearly resolved the six strains into two distinct clades: Pse5, Pse12, Pse16, Pse20, and Pse32 clustered with *P. atacamensis*, while Pse19 clustered with *P. cucumis*. Whole-genome sequencing further revealed that the genome sizes of *P. atacamensis* strains (represented by Pse16) and *P. cucumis* Pse19 were approximately 6,363,964 bp and 5,948,415 bp, respectively (Fig. S5). Despite this species-level divergence, all six strains exhibited similar plant growth-promoting effects, confirming that different *Pseudomonas* species associated with *A. inebrians* can function equivalently in promoting host performance.

## DISCUSSION

Our study integrated ecological inference with functional validation and provided experimental evidence supporting the potential roles of network-identified bacterial taxa under controlled conditions. First, we uncovered that *Epichloë* endophytes reconfigure the bacterial co-occurrence networks in *A. inebrians*, leading to the identification of *Pseudomonas* as a ubiquitous keystone genus. We then bridged network theory with microbiology by revealing that *Pseudomonas* strains abundantly isolated from seed, leaf, and rhizosphere compartments could consistently promote host plant growth and forage quality. This holistic approach, transitioning from network-based discovery to culture-dependent functional assessment, provides a validated paradigm for identifying and harnessing beneficial microbes to improve sustainable agriculture.

### Importance of network analysis in identifying keystone species

Microorganisms interact within complex biological networks through negative, such as predation, positive, such as mutualistic symbiosis, and neutral, such as commensalism, relationships in natural ecosystems (56). Network analysis provides a foundation for identifying and understanding the dynamics and composition of microbial interactions (28, 29, 46). This study revealed a complex pattern of bacterial networks within the seed-borne microbiome, encompassing *A. inebrians* seed endophytic and glume epiphytic communities. These intricate microbial interactions contribute to community stability, favoring beneficial microorganisms that support plant health (15). In contrast, the reduced complexity observed in leaf microbiomes may indicate a shift toward a more specialized microbial community, potentially increasing vulnerability to external disturbances. Notably, *Epichloë* endophyte infection may mitigate the loss of structural complexity by promoting microbial coexistence and stabilizing interactions (57). Proteobacteria and Firmicutes emerged as keystone phyla in both the seedborne and phyllosphere microbiomes of *A. inebrians*. These phyla are frequently associated with seed and leaf microbiomes of various plant species (58, 59) and have been linked to plant growth and resistance (60). In addition, *Pseudomonas* was identified as a common keystone genus across seed-borne and phyllosphere microbiomes of *A. inebrians*, potentially influencing host grass growth and health, such as stress resistance and nutrient absorption, by regulating community stability through mechanisms, such as competition and symbiosis. This finding is consistent with previous research by (41) and (45), which showed that *Pseudomonas*, often abundant in the root and phyllosphere of *A. inebrians*, plays a key ecological role under both salt stress and non-stress conditions.

### Importance of isolating bacteria from multiple niches of *A. inebrians*

In plant–soil–microbial ecology, the composition and diversity of plant-associated microbial communities are a key research focus (61), encompassing seed-associated, phyllosphere, and rhizosphere microbiota (62–64). The community structure within the same plant species can vary significantly across different ecological niches (65). Previous studies have shown that *Epichloë* endophytes regulate plant microbiomes by selectively promoting beneficial microbes (40, 42–44). Notably, our findings are consistent with these observations, showing that *Epichloë* infection significantly increases the abundance of *Pseudomonas*, revealing a potential role in stress resistance. However, unlike previous studies that primarily examined microbial interactions at the individual plant scale, our study reveals how these effects extend to seed dispersal and the phyllosphere microbiome, shedding light on previously underexplored endophytic fungus–microbial interactions. Bacterial strains isolated from seed-associated, phyllosphere, and rhizosphere niches exhibit multifunctional metabolic capabilities, including diazotrophic activity, mineral phosphate mobilization, biosynthesis of phytohormonal regulators, and secondary metabolite-mediated antagonism. These functions collectively contribute to host plant phenotypic plasticity and ecological adaptation (66, 67). Notably, the enrichment of potentially beneficial bacteria, such as *Pseudomonas* and *Bacillus*, has been associated with enhanced seed germination and seedling growth (56, 68).

### Potential mechanisms and applications of *Pseudomonas* as a dominant genus

*Pseudomonas* has a broad spectrum of plant growth-promoting capabilities, operating through various mechanisms, such as nutrient solubilization, pathogen suppression, and the regulation of plant immune responses (69). Future studies should validate these potential roles using metagenomic and functional analyses. Because of the strong plant growth-promoting effects of *Pseudomonas* observed in this study, these strains could be developed into microbial inoculants or biofertilizers to enhance grassland productivity (70). Previous studies have revealed similar applications in crops, such as maize and rice (21, 34, 71), indicating that the benefits of these microorganisms may extend beyond *A. inebrians*. In addition, variations in microbial community structure in response to *Epichloë* endophytic infection reveal that manipulating plant-associated microbiomes could be a viable strategy to improve plant resilience to environmental stress. Future research should explore the feasibility of microbial community engineering in grassland ecosystems. Although this study successfully identified key microbial interactions within the plant, the functional roles of these microbes remain largely unknown. To address this, future studies should use metagenomics and transcriptomics to uncover the genetic and metabolic functions of key taxa, such as *Pseudomonas* (72). Furthermore, because our findings are based on laboratory inoculation experiments, field trials under natural environmental conditions are essential to verify the effectiveness of *Pseudomonas* as a plant growth promoter and assess its potential ecological risks (31). Finally, although this study identified keystone species through network analysis, future studies should integrate single-cell Raman spectroscopy (73) or stable isotope probing to confirm the functional contributions of these taxa *in vivo*. In brief, this is primarily a correlational study. Although key species were identified through network analysis, their exact causal and functional effects still require verification through subsequent studies (for example, removing this key species from synthetic microbial communities).

### Conclusions

**Fig 7:**
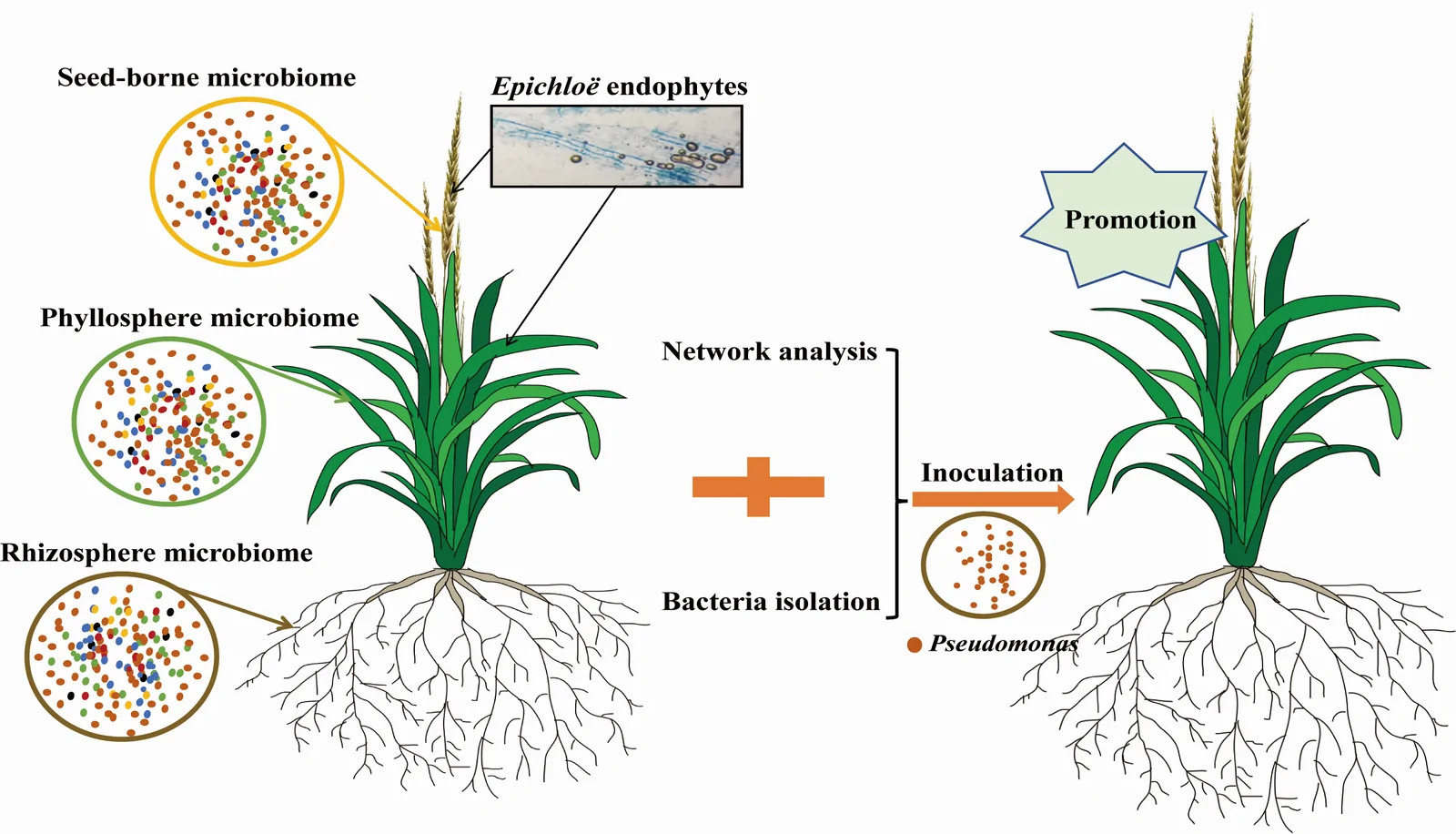
Conceptual diagram illustrating the integration of network analysis and bacterial culturing methods to identify and select keystone *Pseudomonas* strains. These strains were subsequently inoculated into *A. inebrians* to assess the effects on host growth and development.

Collectively, our study reveals that seed-borne bacterial networks exhibit complex, yet tightly interconnected structures. In contrast, phyllosphere bacterial communities display more distinct and cohesive interactions, as assessed through the Avg K index in network analysis. *Pseudomonas* emerged as a common keystone bacterial genus in both seed-associated and phyllosphere microbiomes of *A. inebrians*. This genus accounted for 13, 35, and 33% of all cultivable bacteria in the seed, leaf, and rhizosphere compartments of *A. inebrians*, respectively. Inoculation with *Pseudomonas* strains promoted *A. inebrians* growth, improved forage quality, and enhanced nutrient uptake (Fig. 7). By integrating network theory with functional microbiology, we propose a mechanistic framework in which endophyte-mediated microbiome remodeling enriches keystone taxa that synergistically enhance plant performance. Our findings not only elucidate plant–microbe interactions from a niche-specific perspective but also highlight *Pseudomonas* strains, such as Pse16 and Pse20, as promising candidates for the development of targeted biofertilizers, providing sustainable solutions to improve forage productivity in semi-arid ecosystems. Through whole-genome sequencing, we have now precisely determined the species identity of the six key *Pseudomonas* strains used in this study (e.g., *P. atacamensis *and *P. cucumis*), providing a solid taxonomic foundation for interpreting their functional effects. In future research, precise genomic-level identification and in-depth exploration of the mechanisms of these strains with significant bioprospecting functions is a crucial next step. In addition, synthetic microbial communities should be constructed, and their role in regulating microbiota balance should be functionally verified by targeting the removal and addition of key species.

## Data Availability

The BioProject accession numbers for the seed-borne and phyllosphere bacterial data sets are PRJNA865502 (44) and PRJNA795658 (43), respectively. All data generated or analyzed during this study are included in this article.

## SUPPLEMENTAL MATERIAL

Supplemental material msystems.00563-26-s0001.pdf Supplemental table captions; Tables S8 to S10; Fig. S1 to S5.

Supplemental tables msystems.00563-26-s0002.xlsx Tables S1 to S7 and S11.

ASM does not own the copyrights to Supplemental Material that may be linked to, or accessed through, an article. The authors have granted ASM a non-exclusive, world-wide license to publish the Supplemental Material files. Please contact the corresponding author directly for reuse.
