# Supplementary material for: Unlocking the power of Pseudomonas: network-guided isolation enhances plant growth on Achnatherum inebrians
Source: mSystems. 2026 Sep 2;11(9):e00563-26. doi: 10.1128/msystems.00563-26 (PMC13595936; doi:10.1128/msystems.00563-26)
Supplement: Supplementary file 1 [file msystems.00563-26-s0001.pdf]

Supplement materials

Table S1. Detailed numbers of module hubs and connectors of bacterial networks from SEpI, SEpF, SEnI, SEnF, GEpI to GEpF.

Table S2. Detailed numbers of module hubs and connectors of bacterial networks from LEpI, LEpF, LEnI to LEnF.

Table S3. Spearman's correlation networks between seed-borne keystone splecies and alpha diversity.

Table S4. Spearman's correlation networks between phyllosphere keystone splecies and alpha diversity.

Table S5. Detailed numbers of isolated bacteria from SEpI, SEpF, SEnI, SEnF, GEpI to GEpF.

Table S6. Detailed numbers of isolated bacteria from LEnI, LEnF, LEpI to LEpF.

Table S7. Detailed numbers of isolated bacteria from REI to REF.

Table S8. Relative abundance of bacteria in *A. inebrians* seed at the phylum and genus level.

Table S9. Relative abundance of bacteria in *A. inebrians* leaf at the phylum and genus level.

Table S10. Relative abundance of bacteria in *A. inebrians* rhizosphere at the phylum and genus level.

Table S11. A complete list of all 273 *Pseudomonas* isolates from seed, leaf and rhizosphere soil of *A. inebrians*, including their strain id, species and isolation source.

Table S8. Relative abundance of bacteria in *A. inebrians* seed at the phylum and genus level.

Phylum	Genus	SEnI	SEnF	SEpI	SEpF	GEpI	GEpF	F	P
Proteobacteria	*Acinetobacter*	10.7±1.19e	22.28±1.8d	41.87±0.44a	37.39±0.5b	27.83±0.41c	37.59±0.87b	136.784	0
Proteobacteria	*Brevundimonas*	28.09±0.58a	18.98±0.58b	18.67±0.56b	12.11±0.53c	19.67±0.47b	11.3±0.35c	138.063	0
Bacteroidetes	*Chryseobacterium*	23.95±3.47a	20.2±0.76ab	15.93±0.16bc	12.05±0.1c	14.75±0.45c	15.43±0.84bc	8.141	0.001
Bacteroidetes	*Sphingomonas*	3.77±0.79c	0.8±0.4d	6.7±0.53b	10.46±0.27a	7.01±0.33b	9.73±0.58a	40.267	0
Proteobacteria	*Pseudomonas*	21.33±0.88a	15.47±0.29b	12.07±0.52c	11.67±0.67c	12±1.53c	6.67±0.88d	30.506	0
Proteobacteria	*Prolinoborus*	3.99±0.45b	1.93±1.1bc	0.77±0.38c	0±0d	12.64±0.37a	0±0d	114.007	0
Bacteroidetes	*Sphingobacterium*	3±3b	13.35±0.88a	1.13±0.66b	0±0b	0±.b	11.69±0.3a	16.065	0
Firmicutes	*Bacillus*	0.5±0.36b	3.75±0.71a	0.77±0.38b	0±0b	4.43±0.2a	0±0b	28.771	0
Proteobacteria	*Microbacterium*	1±0.58b	0.87±0.63b	0.13±0.09b	4.07±0.49a	0±0b	0±0b	14.961	0
Bacteroidetes	*Sphingobium*	0.33±0.33b	1.23±0.67b	0±0b	0±0b	0±0b	7.59±1.78a	14.638	0
Bacteroidetes	*Novosphingobium*	3.84±0.26a	0.77±0.38b	0.67±0.33b	0±0b	0±0b	0±0b	109.302	0.002
Proteobacteria	*Aerococcus*	0.77±0.62	0.33±0.2	1.94±1.24	0±0	0±0	0±0	2.567	0.089
Bacteroidetes	*Flavobacterium*	0±0b	0.03±0.03b	0±0b	12.26±1.1a	1.67±0.85b	0±0b	74.788	0

Table S9. Relative abundance of bacteria in *A. inebrians* leaf at the phylum and genus level.

Phylum	Genus	LEnI	LEnF	LEpI	LEpF	F	P
Bacteroidetes	*Chryseobacterium*	4.4±0.3b	3.37±0.34c	5.73±0.2a	5.52±0.31a	14.27	0.00
Firmicutes	*Aerococcus*	11.1±0.3c	15.94±0.39a	12.94±0.15b	12.81±0.88b	15.539	0.001
Firmicutes	*Bacillus*	17.05±0.08a	11±0.06c	16.38±0.61ab	15.37±0.43b	52.24	0.00
Proteobacteria	*Acinetobacter*	12.35±0.85	10.18±0.42	10.1±0.46	9.52±1.05	2.79	0.11
Proteobacteria	*Massilia*	10.96±0.86b	12.79±0.51ab	12.77±0.33ab	13.5±0.64a	3.11	0.09
Proteobacteria	*Pseudomonas*	27.27±0.18a	23.47±0.27b	28.64±0.3a	27.7±0.92a	19.97	0.00
Actinobacteria	*Micrococcus*	0.57±0.07c	3.26±0.07a	1.17±0.03b	1.11±0b	537.68	0.00
Actinobacteria	*Zhihengliuella*	0.63±0.09	1.41±0.16	1.73±0.37	1.69±0.65	1.78	0.23
Flavobacteria	*Kaistella*	0.34±0.09b	1.02±0.04b	2.53±0.47a	2.41±0.64a	7.11	0.01
Firmicutes	*Enterococcus*	2.16±0.22	1.17±0.03	1.03±0.03	3.22±1.07	3.44	0.07
Firmicutes	*Lactococcus*	0.52±0.06b	1.23±0a	1.22±0.12a	1.1±0.05a	23.00	0.00
Proteobacteria	*Acidovorax*	9.99±0.05a	9.64±0.28a	1.32±0.11b	1.4±0.35b	438.91	0.00
Proteobacteria	*Cellvibrio*	0.84±0.33b	1.76±0.09a	1.19±0.04ab	1.07±0.04b	4.95	0.03
Proteobacteria	*Ralstonia*	0.89±0.34	1.03±0.03	1.04±0.04	1.93±0.55	2.17	0.17
Proteobacteria	*Stenotrophomonas*	0.94±0.29	2.73±0.32	2.21±0.58	1.66±0.32	3.78	0.06

Table S10. Relative abundance of bacteria in *A. inebrians* rhizosphere at the phylum and genus level.

Phylum	Genus	EI	EF	F	P
Proteobacteria	*Acinetobacter*	10.44±0.32	10.64±0.32	0.19	0.69
Proteobacteria	*Brevundimonas*	3.64±0.32b	4.86±0.27a	8.41	0.04
Bacteroidetes	*Chryseobacterium*	7.24±0.32b	21.64±0.32a	1000.77	0.00
Proteobacteria	*Pseudomonas*	41.33±0.67a	26.33±1.76b	63.28	0.00
Proteobacteria	*Stenotrophomonas*	8.36±0.33a	5.49±0.33b	38.95	0.00
Firmicutes	*Bacillus*	19.35±0.33a	9.35±0.33b	472.84	0.00
Firmicutes	*Arthrobacter*	0.5±0.18	0.19±0.1	2.27	0.21
Actinobacteria	*Brevibacterium*	0.46±0.07b	3.54±0.23a	169.54	0.00
Proteobacteria	*Erwinia*	0.06±0.06b	3.2±0.04a	2299.78	0.00
Proteobacteria	*Leclercia*	0.77±0.06b	2.23±0.11a	137.76	0.00
Proteobacteria	*Pantoea*	2.6±0.31a	0.9±0.13b	26.51	0.01
Actinobacteria	*Paenarthrobacter*	2.98±0.57	2.85±0.6	0.03	0.88
Proteobacteria	*Phyllobacterium*	1.12±0.1b	3.05±0.58a	10.70	0.03
Proteobacteria	*Rhizobium*	1.17±0.92a	2.24±0.62a	0.93	0.39

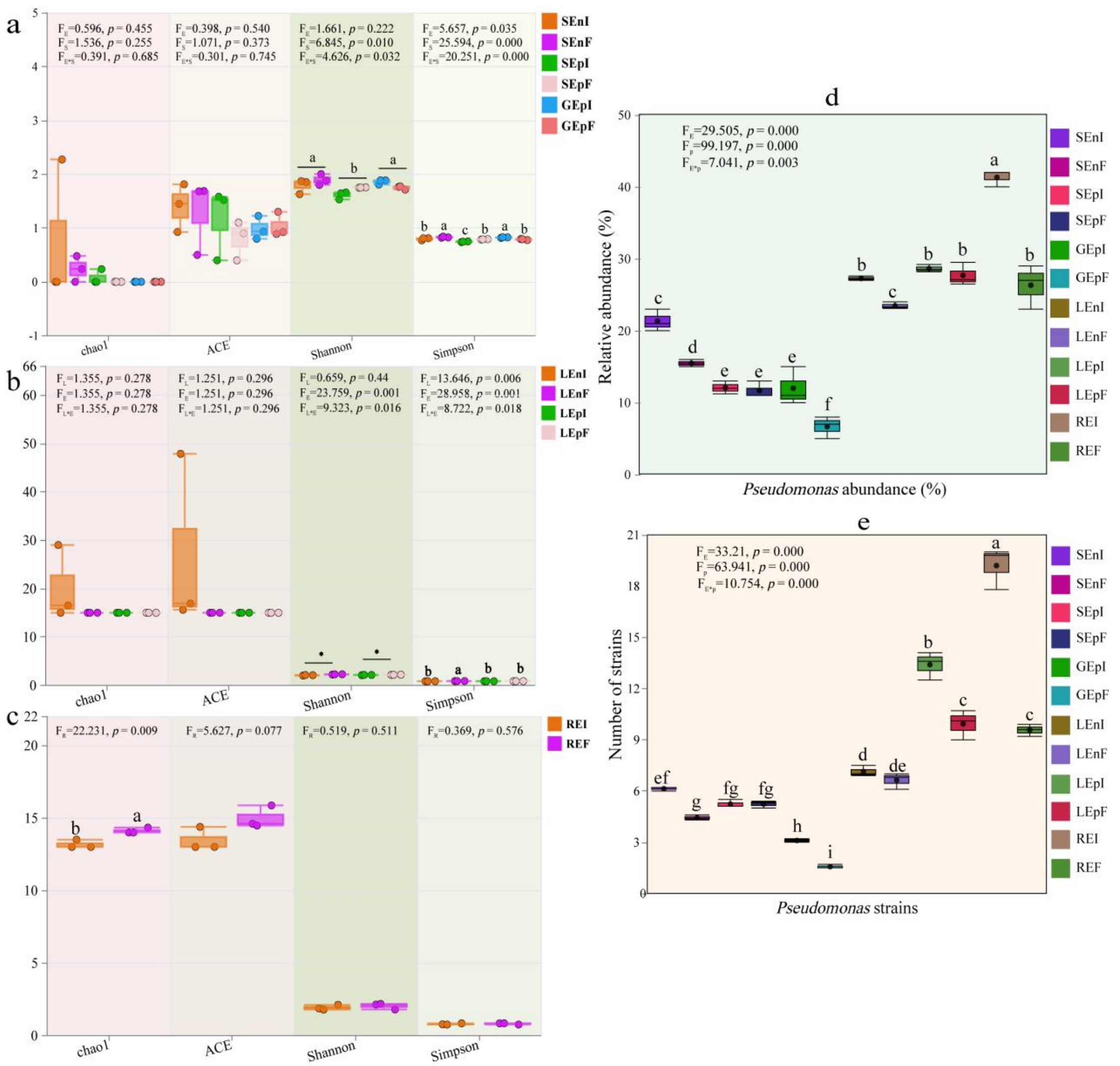

Figure S1. Alpha diversity and *Pseudomonas* abundance in *A. inebrians* seed, leaf, and rhizosphere. a Alpha diversity analysis of *A. inebrians* seed using Chao1, ACE, Shannon, and Simpson indices. **b** Alpha diversity analysis of *A. inebrians* leaf using Chao1, ACE, Shannon, and Simpson indices. **c** Alpha diversity analysis of *A. inebrians* rhizosphere using Chao1, ACE, Shannon, and Simpson indices. **d** Relative abundance of *Pseudomonas* in seed, leaf, and rhizosphere of *A. inebrians*. **e** Number of isolated *Pseudomonas* strains from seed, leaf, and rhizosphere of *A. inebrians*. E+: EI; E-: EF; Pse16, Pse20, Pse19, Pse32, Pse12, and Pse5 represent inoculation with different *Pseudomonas* strains; E: *Epichloë* infection status; P: *Pseudomonas* inoculation in EI and EF *A. inebrians*. Different lowercase letters indicate significant differences (P<0.05) among different treatments. Asterisks (*) represent significant differences between EI and EF.

a

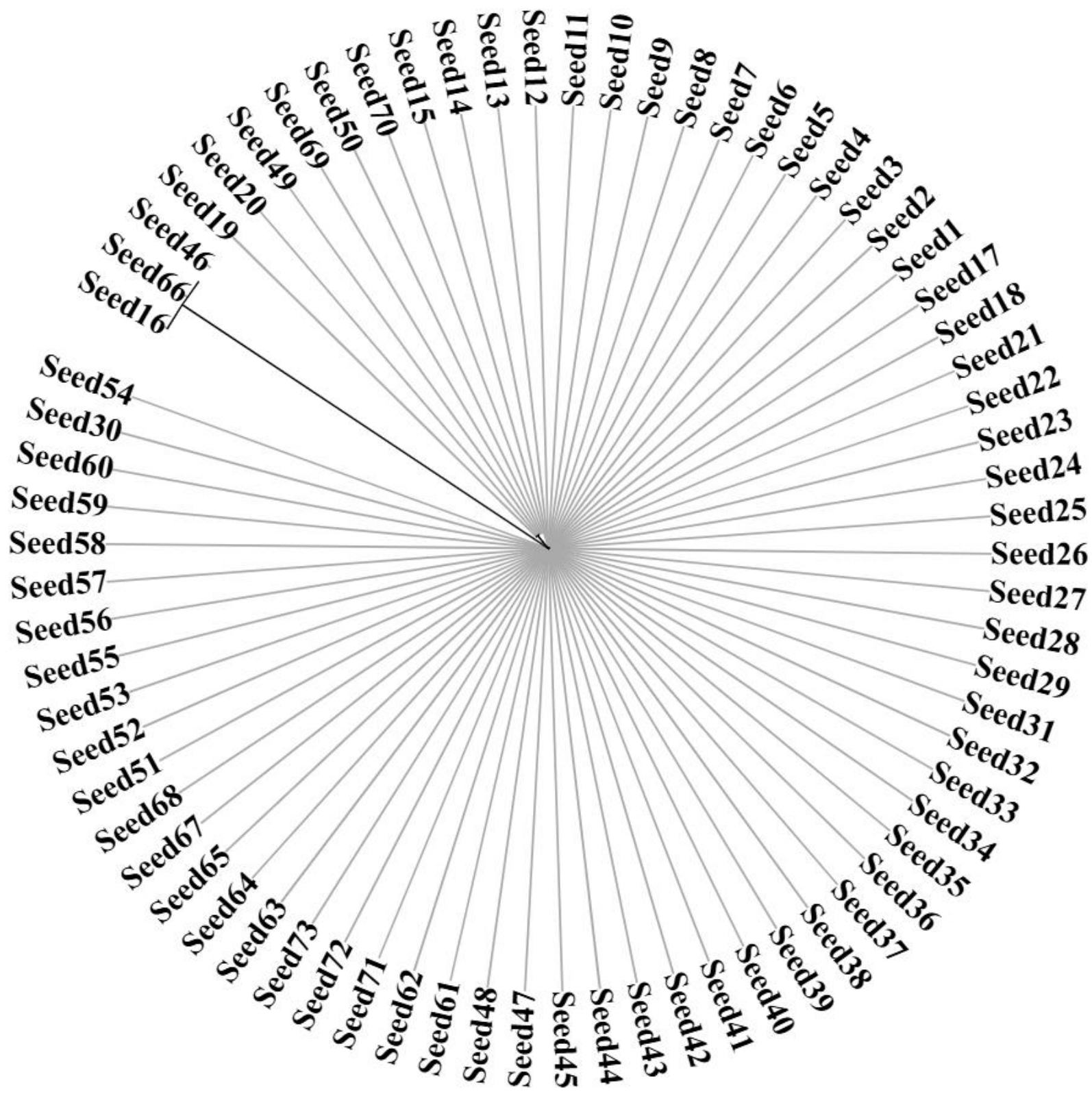
Seed12
Seed11
Seed10
Seed9
Seed8
Seed7
Seed6
Seed5
Seed4
Seed3
Seed2
Seed1
Seed17
Seed18
Seed21
Seed22
Seed23
Seed24
Seed25
Seed26
Seed27
Seed28
Seed29
Seed31
Seed32
Seed33
Seed34
Seed35
Seed36
Seed37
Seed38
Seed39
Seed40
Seed41
Seed42
Seed43
Seed44
Seed45
Seed47
Seed48
Seed61
Seed62
Seed71
Seed72
Seed73
Seed63
Seed64
Seed65
Seed67
Seed68
Seed51
Seed52
Seed53
Seed55
Seed56
Seed57
Seed58
Seed59
Seed60
Seed30
Seed54
Seed16
Seed66
Seed46
Seed19
Seed20
Seed49
Seed69
Seed50
Seed70
Seed15
Seed14
Seed13

b

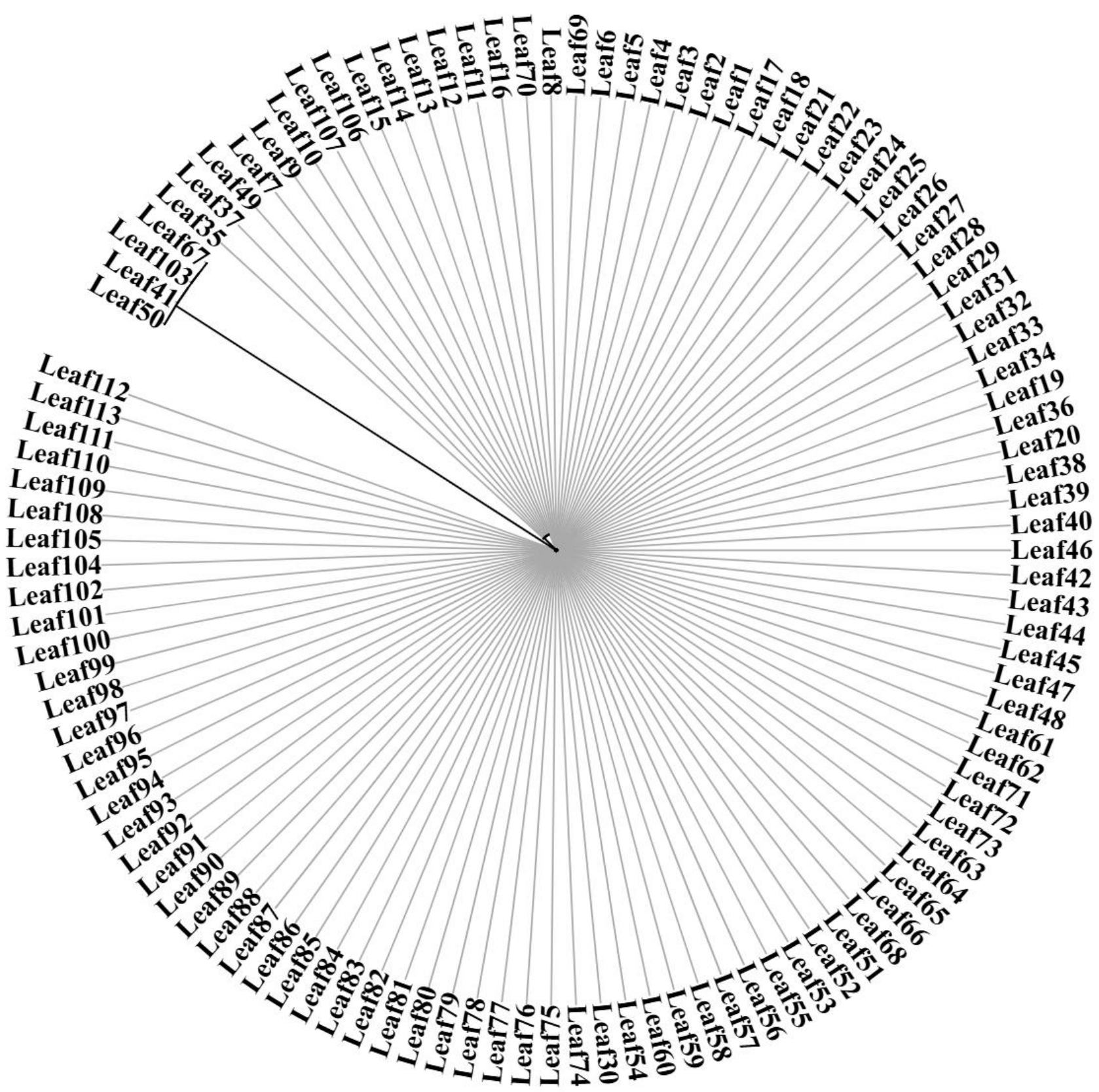
Leaf8
Leaf69
Leaf6
Leaf5
Leaf4
Leaf3
Leaf2
Leaf1
Leaf17
Leaf18
Leaf21
Leaf22
Leaf23
Leaf24
Leaf25
Leaf26
Leaf27
Leaf28
Leaf29
Leaf31
Leaf32
Leaf33
Leaf34
Leaf19
Leaf36
Leaf20
Leaf38
Leaf39
Leaf40
Leaf46
Leaf42
Leaf43
Leaf44
Leaf45
Leaf47
Leaf48
Leaf61
Leaf62
Leaf71
Leaf72
Leaf73
Leaf63
Leaf64
Leaf65
Leaf66
Leaf68
Leaf51
Leaf52
Leaf53
Leaf55
Leaf56
Leaf57
Leaf58
Leaf59
Leaf60
Leaf54
Leaf30
Leaf74
Leaf75
Leaf76
Leaf77
Leaf78
Leaf79
Leaf80
Leaf81
Leaf82
Leaf83
Leaf84
Leaf85
Leaf86
Leaf87
Leaf88
Leaf89
Leaf90
Leaf91
Leaf92
Leaf93
Leaf94
Leaf95
Leaf96
Leaf97
Leaf98
Leaf99
Leaf100
Leaf101
Leaf102
Leaf104
Leaf105
Leaf108
Leaf109
Leaf110
Leaf111
Leaf113
Leaf112
Leaf50
Leaf41
Leaf103
Leaf67
Leaf35
Leaf37
Leaf49
Leaf7
Leaf9
Leaf10
Leaf107
Leaf106
Leaf15
Leaf14
Leaf13
Leaf12
Leaf11
Leaf16
Leaf70

c

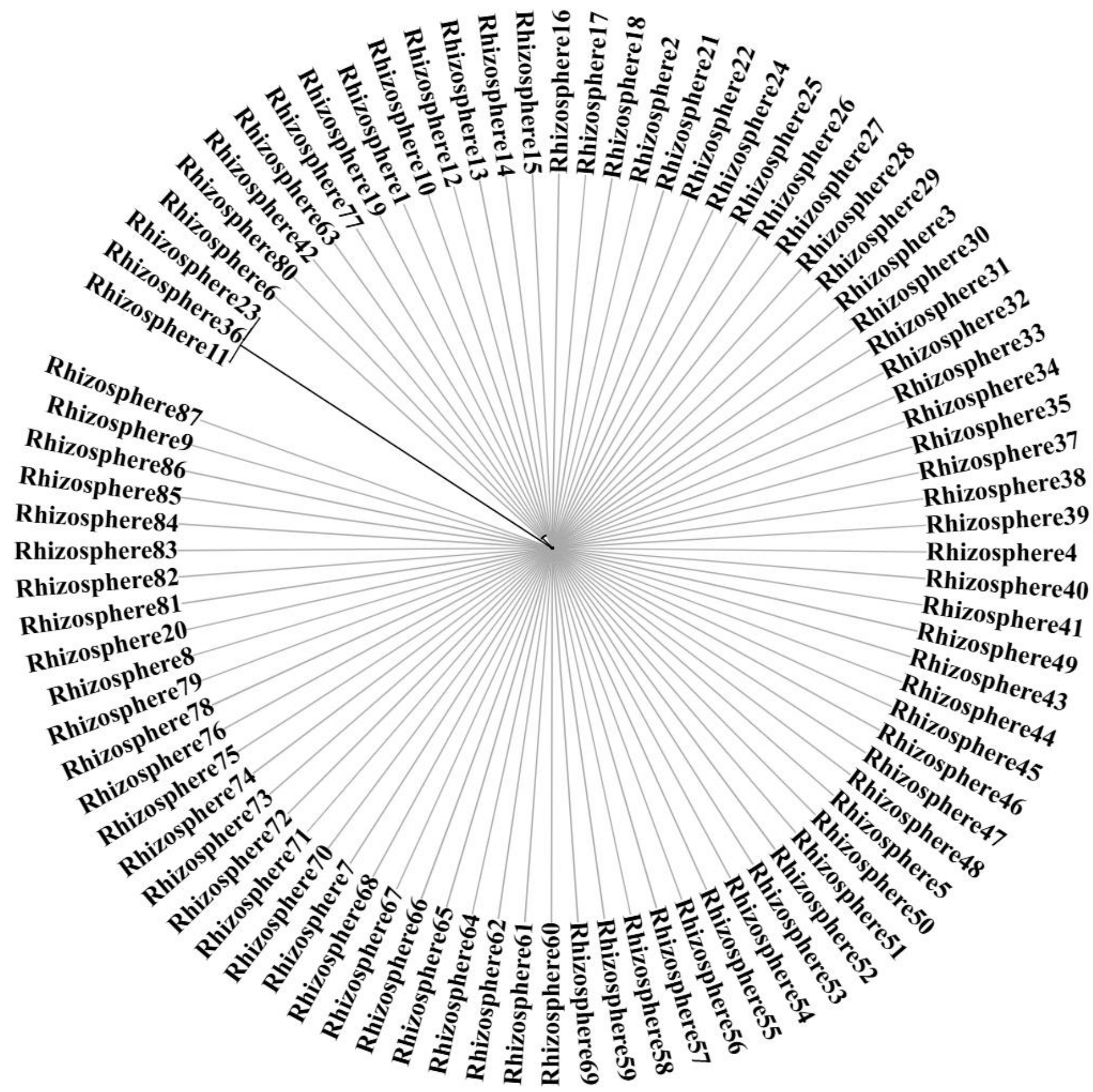

Figure S2. Neighbour-joining (NJ) phylogenetic tree analysis of *Pseudomonas* bacteria isolated from *A. inebrians* seed (a), leaf (b), and rhizosphere soil (c). (Seed1-Seed73 represented 73 *Pseudomonas* strains isolated from *A. inebrians* seed, Leaf1-Leaf113 represented 113 *Pseudomonas* strains isolated from *A. inebrians* leaf, Rhizosphere1-Rhizosphere87 represented 87 *Pseudomonas* strains isolated from *A. inebrians* rhizosphere soil).

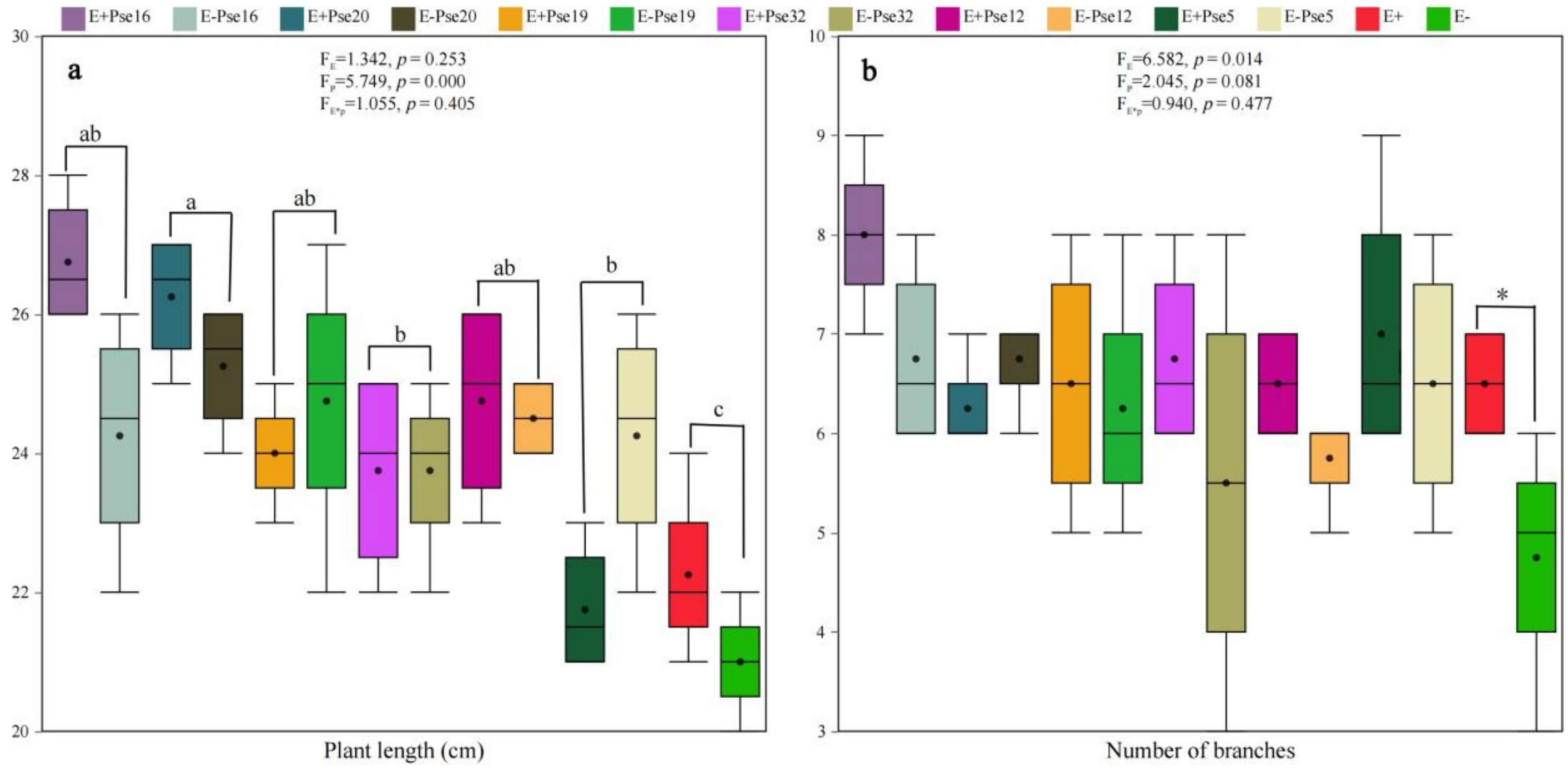

Figure S3. **The effect of inoculating *Pseudomonas* strains on the plant length and number of branches of endophyte-infected *A. inebrians* and endophyte-free *A. inebrians*. a** plant length, **b** number of branches. (E+: *Epichloë* endophyte-infected, E-: *Epichloë* endophyte-free, Pse16, Pse20, Pse19, Pse32, Pse12 and Pse5 represented inoculating different *Pseudomonas* strains, E: *Epichloë* endophyte infection status, P: inoculating *Pseudomonas* on EI and EF *A. inebrians*, These small letters indicated mean significant difference at P<0.05 among different inoculations. The * represented there is a significant difference between EI and EF.)

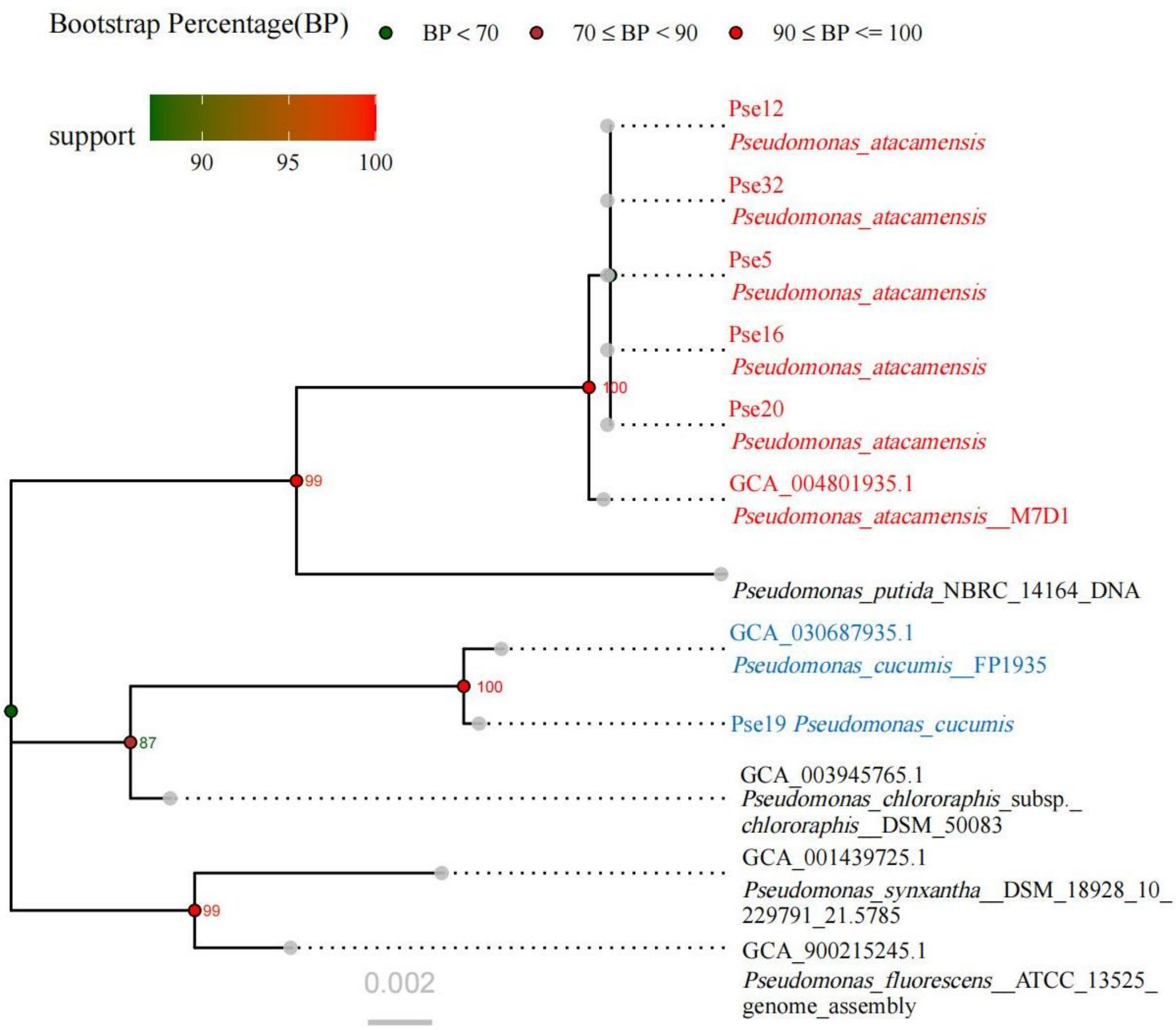

Figure S4. The core-genome phylogenetic tree clearly resolved the six strains into two distinct clades: Pse5, Pse12, Pse16, Pse20, and Pse32 clustered with the type strain, while Pse19 clustered with *P. cucumis*.

Note: *P. cucumis* FP1935^T (GenBank assembly accession GCA_030687935.1), *P. atacamensis* M7D1^T (GCA_004801935.1), *P. putida* NBRC 14164^T (GCA_000412675.1), *P. fluorescens* ATCC 13525^T (GCA_900215245.1), *P. synxantha* DSM 18928^T (GCA_001439725.1), and *P. chlororaphis* subsp. *chlororaphis* DSM 50083^T (GCA_003945765.1).

a

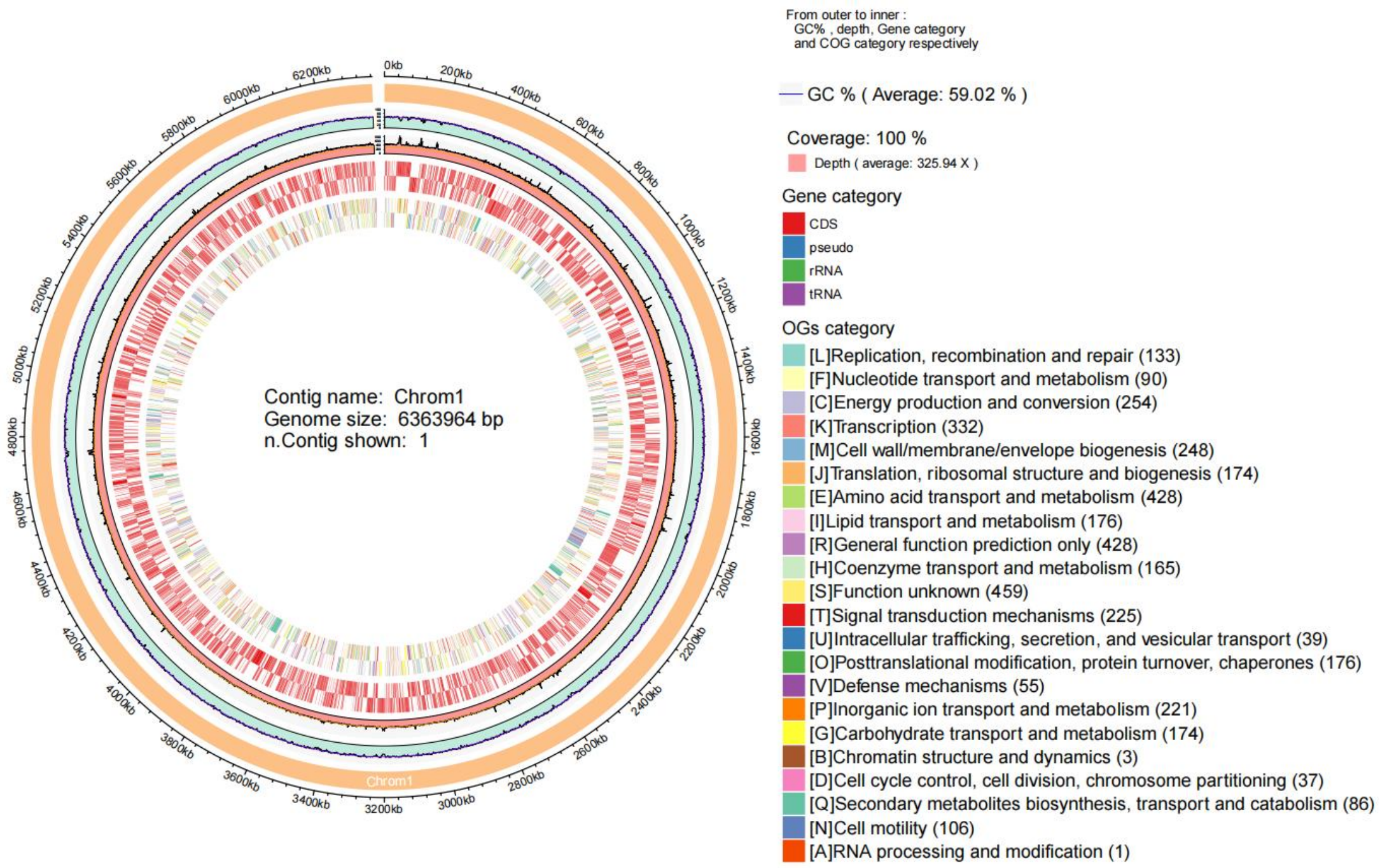

b

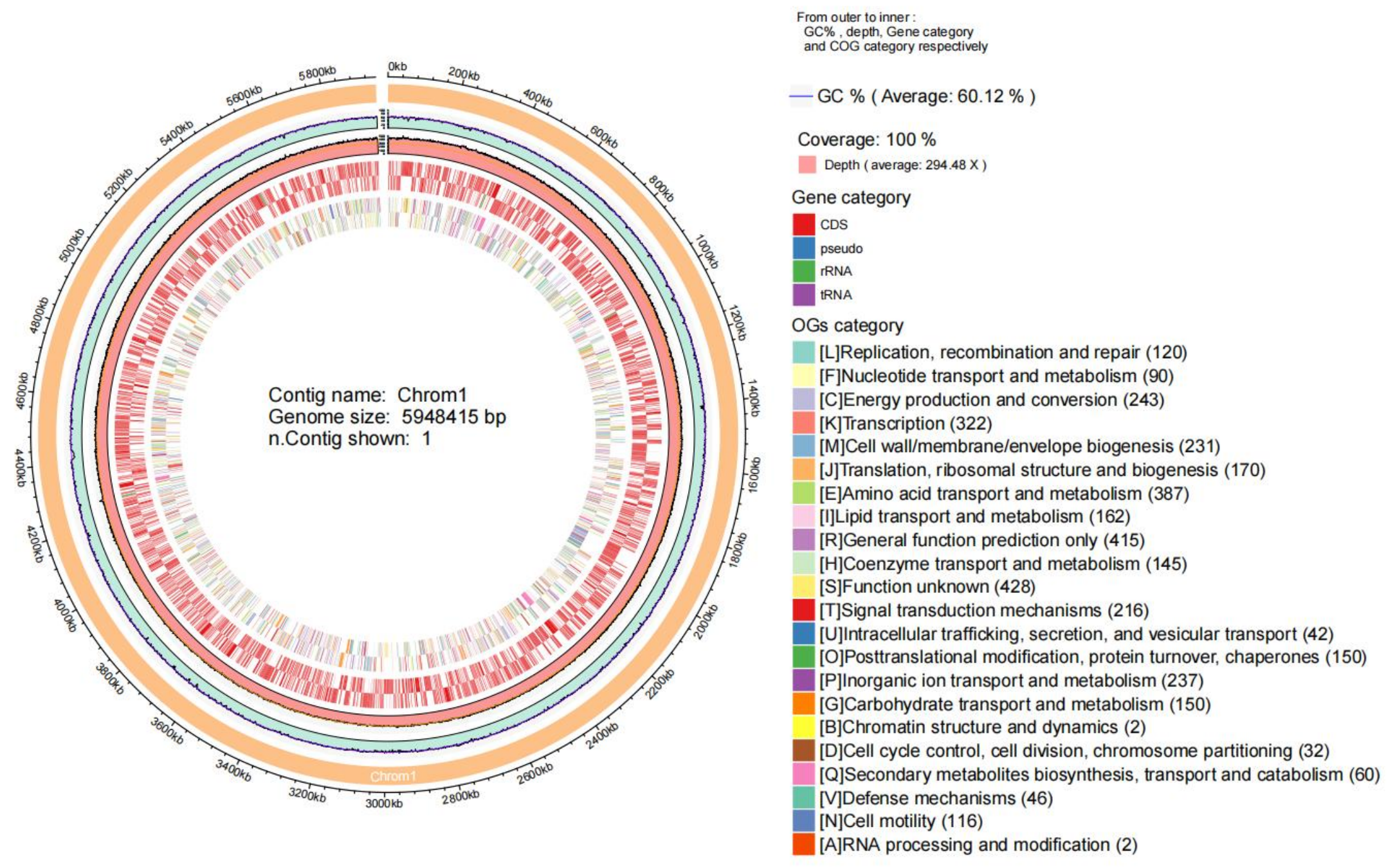

Figure S5. The circos diagram of the genome shows that the circles are presented from the outside to the inside, showing GC content, sequencing depth, gene element display, and COG function display. a (Pse5, Pse16, Pse20, Pse32 and Pse12 were identified as *P. atacamensis*), b (Pse19 was identified as *P. cucumis*).
